# CIZ1 regulates G1 length and the CDK threshold for initiation of DNA replication to prevent DNA replication stress

**DOI:** 10.1093/nar/gkag878

**Published:** 2026-09-12

**Authors:** James Tollitt, Marcus K Preedy, Tiernan Briggs, Sarah L Allinson, Christopher J Staples, Jason L Parsons, Richard L Mort, Nikki A Copeland

**Affiliations:** Biomedical and Life Sciences, Faculty of Health and Medicine, Lancaster University, Bailrigg, Lancaster, LA1 4YQ, United Kingdom; Biomedical and Life Sciences, Faculty of Health and Medicine, Lancaster University, Bailrigg, Lancaster, LA1 4YQ, United Kingdom; Biomedical and Life Sciences, Faculty of Health and Medicine, Lancaster University, Bailrigg, Lancaster, LA1 4YQ, United Kingdom; Biomedical and Life Sciences, Faculty of Health and Medicine, Lancaster University, Bailrigg, Lancaster, LA1 4YQ, United Kingdom; North West Cancer Research Institute, North Wales Medical School, Bangor University, Bangor, Gwynedd LL57 2UW, United Kingdom; Institute of Cancer and Genomic Sciences, College of Medical and Dental Sciences, University of Birmingham, Edgbaston, Birmingham B15 2TT, United Kingdom; Biomedical and Life Sciences, Faculty of Health and Medicine, Lancaster University, Bailrigg, Lancaster, LA1 4YQ, United Kingdom; Biomedical and Life Sciences, Faculty of Health and Medicine, Lancaster University, Bailrigg, Lancaster, LA1 4YQ, United Kingdom

## Abstract

Eukaryotic cell division is regulated by CDK activity that must reach critical CDK threshold levels to progress through cell cycle stages. In low-mitogen, low-CDK environments, cells exit the cell cycle into a non-proliferative quiescent state, G0, that plays essential roles in stem cell maintenance and cellular homeostasis. CIZ1 regulates cell cycle and epigenetic programmes, and CIZ1 ablation promotes genomic instability after release from quiescence. Here, we show that CIZ1 contributes to mechanisms that temporally regulate cell cycle transitions in post-quiescent cells. *CIZ1^−/−^* (CIZ1 KO) fibroblasts re-entering the cell cycle from quiescence have reduced G1 phase and cell cycle length, mediated by increased intracellular CDK activity and early restriction point bypass via G1/S cyclin overexpression. In addition, *CIZ1^−/−^* cells are deficient in cyclin A chromatin binding and require increased CDK activity to initiate DNA replication, leading to DNA replication stress. Importantly, ectopic expression of *CIZ1* or addition of recombinant CIZ1 reinstates the CDK threshold for initiation of DNA replication, reversing DNA replication stress and increasing replication fork rates. These data suggest that in post-quiescent cells, CIZ1 determines the threshold CDK activity required for the G1/S transition to prevent DNA replication stress.

## Graphical Abstract

**Graphical Abstract:**
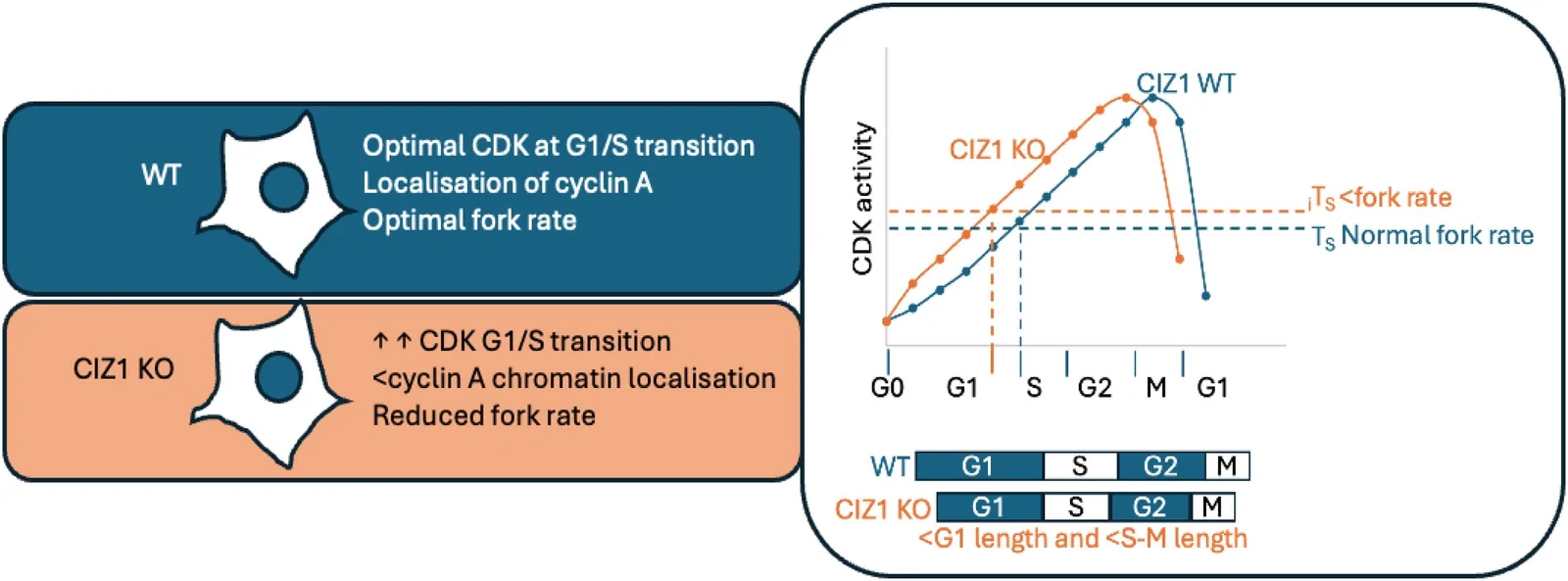

## Introduction

Eukaryotic cells use a reversible cell cycle exit, quiescence or G0, to maintain a lifelong pool of cells that can proliferate in response to mitogenic signalling to maintain tissue homeostasis [1, 2]. Quiescence (G0) is essential to prevent replicative senescence in young organisms and also contributes to developmental programmes, stem cell maintenance, and healthy aging [2]. Metazoan cells exit the cell cycle and enter quiescence *in vitro* in low-mitogen environments or at high cell density through contact inhibition [3]. The ability of cells to exit proliferation cycles into a quiescent state (G0) also has implications for therapeutic responses in cancer, where non-proliferative populations of cells are more resistant to cancer therapies [4–6]. Therefore, a more complete understanding of the regulatory processes that facilitate quiescence entry and exit are required.

Here, we investigate the role of CIP1 Interacting Zinc finger protein 1 (CIZ1) in cycling and post-quiescent fibroblasts. CIZ1 has multifaceted roles, contributing to epigenetic regulation [7], maintenance of X-chromosome repression and localization [8, 9], oncogenic transcriptional programs [10, 11], and cell cycle regulation [12, 13]. CIZ1 is a CDK sensor that promotes cell cycle progression and facilitates initiation of DNA replication [14–16]. CIZ1 is a non-essential gene [8, 17]. However, CIZ1-null mice have defects in neurological function and increased DNA damage responses [18]. CIZ1 contributes to epigenetic maintenance in primary fibroblasts [19] and increases the frequency of lymphoblastic tumour formation [8, 17], potentially revealing a tumour suppressor function. CIZ1 also regulates the G1/S transition through interactions with both cyclin–CDK complexes and CDK inhibitor protein p21 [14, 15, 20]. CIZ1 promotes the G1/S transition through its interactions with cyclin E–CDK2 and cyclin A–CDK2 through localization of their activity to chromatin to enhance the efficiency of the initiation phase of DNA replication [14], and this interaction is regulated post-translationally through CDK-mediated phosphorylation of CIZ1 [15].

CIZ1 ablation revealed defects in cell cycle re-entry from quiescence that underpin cellular transformation [21]; however, the precise role for CIZ1 in maintaining genome integrity in proliferation–quiescence cycles is not fully understood. Quiescence is regulated by extracellular, intercellular, and intracellular signalling [2, 22, 23]. The regulation of the proliferation or quiescence decision is mediated by extracellular mitogen levels and intrinsic CDK activity levels inherited from the mother cell. There are exit points from the cell cycle into a non-replicative quiescent state in mitosis and G1 phase of the cell cycle [24, 25]. Intrinsic CDK activity plays a key role in the proliferation–quiescence decision and dictates the length of G1 phase, as bifurcation of CDK activity dictates whether cells rapidly exit G1 phase or at lower CDK levels enter a prolonged G1 state [25]. Alterations in mitogenic signalling are integrated into cell cycle proliferation decisions, providing a memory of mitogen exposure through alteration of cyclin D levels [26]. Intracellular CDK activity is regulated by the balance of CDK activity and CDK inhibitor protein levels that provide a molecular memory of genotoxic stress from previous cell cycles [25, 27–30].

In mammalian cells, the restriction point (R) determines the phase of G1 at which cells no longer require mitogens to complete the cell cycle [31–34]. The mechanistic basis of the restriction point involves the integration of extracellular and intracellular signalling, including transcriptional regulation via the RB–E2F pathway and intracellular CDK activity [23, 35]. There is a precise CDK threshold at which cells bypass R and progress through the cell cycle, and the level of intracellular CDK activity in G1 phase accurately predicts the cells that will bypass R in the absence of mitogenic signalling [36]. Intracellular CDK activity controls the relative depth of quiescence, with low-CDK-activity cells leading to a deeper quiescent state or potentially senescence, whereas higher-CDK-activity cells are more likely to rapidly exit quiescence [23, 37].

The murine CIZ1 KO models show an increased risk of cellular transformation and genomic instability linked to alterations in the epigenetic landscape [17, 19, 21, 38]. Genetic instability is associated with quiescence and proliferation cycles that affected chromatin compaction [21]. It remains to be addressed how CIZ1 KO affects cell cycle regulation and if there are links between cell cycle regulation and the observed genomic instability in CIZ1 KO cells. To address these points, two live cell cycle reporters were produced to monitor cell cycle progression using Fucci(CA) [39, 40] and CDK activity using a fragment of DNA helicase B (DHB) [22, 25]. Here, we show that CIZ1 regulates cell cycle dynamics in post-quiescent cells. Comparison of parental and *CIZ1^−/−^* (CIZ1 KO) cells shows that CIZ1 contributes to mechanisms that regulate G1 length and establish the CDK threshold for G1 exit and initiation of DNA replication. The loss of CIZ1 promotes increased of cyclin E and cyclin A expression and is associated with cyclin A mislocalization. The increased intracellular CDK activity in CIZ1 KO cells has profound effects on restriction point timing and G1 length in post-quiescent cells, ultimately resulting in a DNA replication stress phenotype. Taken together, these data provide insights into the molecular function of CIZ1 in post-quiescent cells and its role in prevention of CDK-mediated DNA replication stress.

## Materials and methods

### Cell culture

Cell culture was performed using aseptic techniques in a laminar flow hood. Mycoplasma testing of cell lines was performed routinely to ensure no contamination. Cell lines used were NIH 3T3 cells (ATCC CRL-1658), NIH 3T3-derived *CIZ1*^−/−^ (hereafter referred to as CIZ1 KO), and CIZ1 KO cells with stably integrated GFP-CIZ1 referred to as CIZ1 add back (CIZ1-AB). Parental cells and CIZ1 KO cells were also produced containing the Fucci(CA) construct [39]. All cells were cultured in Dulbecco’s modified Eagle’s medium (DMEM) low glucose (1g/l glucose) Glutamax II plus pyruvate (Gibco), 10% (v/v) foetal bovine serum (LABTECH), penicillin, streptomycin, glutamine (1×, Gibco) at 37°C. Cells were passaged every 2–3 days to ensure 70% confluency was not exceeded.

### G0 cell synchronization

NIH 3T3 cells were synchronized in G0 by contact inhibition [14, 15, 41]. At confluence, media were changed, and cells were cultured for a further 48 h. Cells were released from G0 by passaging with a minimum 1:4 dilution. Cell cycle synchronization was confirmed by EdU labelling and quantification of S phase entry. In addition, cell cycle kinetics were assessed using Fucci(CA) live-cell imaging to provide complementary cell cycle kinetics data.

### CRISPR-Cas9

Cells were cultured to 70% confluence, and 1 × 10^6^ cells were transfected using a Lonza nucleofector 2B, Kit R, transfection program U-30. CRISPR-Cas9 knockout was performed using the CD4+ gene art plasmid kit (Invitrogen) according to manufacturer’s instructions. Clonal cell lines were DNA-sequenced using nested polymerase chain reaction (PCR), and confirmed frameshifts/nonsense mutations were identified. Subsequently, loss of CIZ1 protein expression was confirmed by western blot.

Two CIZ1 KO clones were produced for each guide RNA (Ciz1 KO1 for 5′ – TTGCTCCTACAGCAGTTGCAGTTTT- 3′, CIZ1 KO1 rev 5′ -TCGAACTGCTGTAGGAGCAACGGTG – 3′) and KO1.2 (CIZ1 KO2 for 5′ – CAAGGTATGGCAGTTCCCCGGTTTT – 3′ and CIZ1 KO1.2 rev 5′ – CGGGGAACTGCCATACCTTGCGGTG – 3′).

### Cloning of DHB:mNeonGreen-P2A-H2B:mScarlet into pB-H1.0-Fucci(CA)-WPRE

The pB-H1.0-Fucci(CA)-WPRE plasmid was a gift from Richard Mort. The pRM14 plasmid, containing DHB:mNeonGreen-P2A-H2B:mScarlet, was a gift from David Matus (Addgene plasmid #163693; http://n2t.net/addgene:163693;  RRID: Addgene_163693) [22]. Restriction digest of pB-H1.0-Fucci(CA)-WPRE (1 μg) was performed using MfeI and PmeI from NEB, following the manufacturer’s protocol. Following digestion, the product was run on a 2% agarose gel containing 0.01% SYBR™ Safe DNA Gel Stain (Thermo Fisher Scientific). The desired 8066-bp band was extracted using the PureLink™ Quick Gel Extraction Kit (Invitrogen) in accordance with the manufacturer’s protocol. DNA was quantified using a Nanodrop One Microvolume UV-Vis Spectrophotometer (Thermo Fisher Scientific) to determine appropriate volumes for the ligation reaction. For the PCR reaction, 0.5 μM of forward and reverse primers (Table 1) was added to 10 ng of pRM14 using Phusion™ Hot Start II DNA Polymerase (Thermo Fisher Scientific) with HF buffer in a DNA Engine® Thermal Cycler (Bio-Rad). Conditions for PCR amplification can be found in Table 2. Following amplification, the PCR product was run on a 2% agarose gel containing 0.01% SYBR™ Safe DNA Gel Stain (Thermo Fisher Scientific). The desired 2263-bp amplicon was extracted using the PureLink™ Quick Gel Extraction Kit (Invitrogen) in accordance with the manufacturer’s protocol. DNA was quantified using a Nanodrop One Microvolume UV-Vis Spectrophotometer (Thermo Fisher Scientific). The DHB:mNeonGreen-P2A-H2B:mScarlet amplicon was ligated into the linearized pB-H1.0-Fucci(CA)-WPRE plasmid through HiFi assembly. The linearized plasmid was incubated with the insert in a 1:2 molar ratio with NEBuilder® HiFi DNA Assembly Master Mix for 1 h at 50°C. The resulting product was then transformed into DH5α competent *Escherichia coli*. Bacteria were plated onto LB plates containing 100 µg/ml of ampicillin and left to grow overnight at 37°C. Colonies were then miniprepped using the PureLink™ Quick Plasmid Miniprep Kit (Invitrogen), and positive colonies were validated through restriction digest with MfeI and AgeI from NEB. Plasmids were nanopore sequenced by Source BioScience Genomics.

**Table 1.** Primers for PCR amplification of DHB:mNeonGreen-P2A-H2B:mScarlet

Name	Sequence
PCR primers	Forward: 5′ ccgatccccgggtaccgggcaattggccgccaccatgacaaatgatgtcacctggagcgaggc-3′
	Reverse: 5′-cttatcgagcggccgagtttaaacgctttaacagagagaagttcgtggctccagagaatcgatttactt-3′

**Table 2.** PCR conditions for Phusion™ Hot Start II DNA Polymerase

Stage	Initial denaturation	Denaturation	Annealing	Elongation	Final	End
		X35 times				
**Temp (°C)**	98	98	60	72	72	12
**Time (s)**	30	10	30	45	300	∞

### Image analysis: DHB CDK sensor analysis

We adapted an ImageJ macro toolset developed by Richard Mort (https://github.com/richiemort79/mitosis_tools/blob/main/Mitosis_Tools.ijm) to calculate cytoplasmic-to-nuclear intensity ratios for each user-defined X–Y position. These ratios were then used to plot cytoplasmic/nuclear values for individual cells at each time point.

### Fucci(CA) cell line generation

Fucci(CA) and pRM14 expressing CDK sensor cell lines were generated using Tandem-Fucci(CA) [39] cloned into a piggyBac-compatible plasmid. Integration was achieved using the piggyBac recombination system [42] using transfection and selection as described earlier.

### Bacterial transformation

Competent Top10 *E. coli* was transformed on ice for 30 min. Plasmid DNA was added and incubated on ice for 30 min. Cells were heat-shocked at 42°C for 45 s and placed back on ice for 2 min. Five hundred microlitres of prewarmed SOC media were added and placed in a shaking incubator at 37°C for 2 h. Outgrowth was plated on prewarmed LB agar plates supplemented with appropriate selection antibiotics.

### Whole-plasmid site-directed mutagenesis

Primers used for site-directed mutagenesis of GFP-CIZ1 and CIZ1-N471-GST to remove the Cy-II box [14] were GGCGAGGCAGGCACAGACACAG and AGCGCTGGCTGGGTCTGGATCTG. PCR reactions followed the QuikChange site-directed mutagenesis kit (Stratagene). PCR reactions were digested with *DpnI* (NEB), and the unmethylated plasmid DNA was used to transform TOP 10 competent *E. coli* cells. Single colonies were cultured, plasmids purified, and mutation confirmed by DNA sequencing (MWG Eurofins).

### Transfection (plasmid DNA and siRNA)

Transfection was performed using the Nucleofector system (Lonza). Transfection Amaxa® Cell Line Nucleofector® Kit R was used with 1 µg of plasmid following recommended procedures. Transfection reagents were equilibrated to room temperature for 30 min prior to transfections. Cells were transfected using program U-30. Cells incubated in fresh media for 48 h, and media plus selection was added for all further passages. For CIZ1 WT and CIZ1 KO cell lines plus Fucci(CA) puromycin was added at 1 mg/ml (Gibco) and CIZ1 AB cells G418 (Gibco) was added at 500 mg/ml. Silencer select verified short interfering RNAs (siRNAs) (Life technologies) were used to deplete: Control siRNA 1 (4390843), *CCNE1* (s63521), *CCNE2* (s63526), and *CCNA2* (s63506). siRNAs were diluted to 100 μM in nuclease-free water and transfected into 3T3 cells using nucleofection Kit R (Lonza) programme U30 using a nucleofector 2C. Cells were harvested for EdU labelling between 14 and 24 h post-release from quiescence, western blot analysis 24 h after release, and qRT-PCR, RNA extracted 24 h post-release. Live-cell imaging was performed from 4 to 50 h post-transfection using WT and KO pRM14 cells expressing a DHB fragment as a CDK sensor.

### Live cell imaging—Fucci(CA)

Live cell imaging of Fucci(CA) cells was performed using a Zeiss LSM880 confocal microscope with an environmental chamber maintaining a constant temperature of 37°C and providing 5% CO_2_ in air. Cells were plated on a CellVis 24-well imaging plate, and images were acquired at 15-min intervals. For cell cycle analysis, data were collected for 60 h and analysed using the Fiji distribution of ImageJ using the Fucci_Tools.ijm plugin (https://github.com/richiemort79/fucci_tools) [40]. For serum removal experiments, serum-containing media were removed, cells were washed in phosphate buffered saline (PBS), and serum-free media were added. Images were taken from 17 h post-release until 26 h.

### Live cell imaging—DHB1 sensor

3T3 cells were synchronized by contact inhibition for 48 h, and cells were released by 1:4 dilution onto a CellVis 24-well imaging plate. Once cells had adhered, imaging was performed using an LSM 880 confocal microscope with environmental controls at 37°C and 5% CO_2_. The pinhole was set at 3 Airy units using a Plan Apochromat 20×/0.8 M27 objective. mScarlet-Histone 2B (excitation laser 561 nm/emission detection 574–712 nm) and mNeon Green-DHB (excitation 488 nm laser, emission 493–556 nm) were detected independently using the average of 2 images and speed 7. A 2 × 2 tile was used for each time point at 15-min intervals for 200 cycles for each well image. Data are generated from three independent biological replicates, with a total of 50 cells. To ensure that all cells were from the first cell cycle after mitosis, cells were monitored to ensure only a single mitosis was observed. Cells that had previously divided after release from quiescence were excluded from the analysis.

Fluorescent intensity was determined using the Fiji distribution of ImageJ using the Nikki_Tools.ijm pluginhttps://zenodo.org/account/settings/github/repository/richiemort79/mitosis_cyto_nuc. This tool uses the Histone 2B-mScarlet signal to identify the nucleus and generate a mask for detection of nuclear fluorescence of DHB-mNeon Green. A two-pixel radius was used to exclude nuclear fluorescence, and a 5-pixel boundary was used to detect cytoplasmic DHB-mNeon Green signal. The data are shown as the ratio of cytoplasmic to nuclear fluorescence. The data were smoothed by using an average of three frames and standardized to the maximum fluorescence signal detected typically just prior to mitosis. Data were averaged for the population fluorescence. For threshold data, 25% and 50% of the maximum fluorescence signals were selected to determine of there was a difference in intrinsic CDK activity.

### Immunoblotting

Cells were harvested in PBS with 1 mM Dithiothreitol (DTT), 1 mM phenylmethylsulfonyl fluoride (PMSF), and phosphatase inhibitors (10 mM NaF, 5 mM sodium orthovanadate, 5 mM beta-glycerophosphate, and 5 mM sodium pyrophosphate). Chromatin fractionation was performed by addition of Triton X-100 on ice for 5 min, followed by 14 000 × *g* centrifugation (chromatin pellet, cytosol supernatant). The supernatant was removed, and an equal volume of 4× sodium dodecyl sulphate–polyacrylamide gel electrophoresis (SDS–PAGE) loading buffer was added before boiling. Chromatin samples were resuspended up to 1× with SDS loading buffer. Samples were analysed by electrophoresis on SDS–PAGE gels (8%, 10%, or 15% v/v) and transferred to a PVDF membrane by semi-dry transfer. Membranes were blocked in 1% w/v bovine serum albumin (BSA) in TBS-Tween (0.1%) (TBS-T). Primary antibodies were incubated overnight at 4°C (anti-cyclin A C4710, Sigma–Aldrich 1:1000; cyclin E HE-12 Abcam 1:500; anti-actin A1978 Sigma–Aldrich, 1:2000, anti-H3 (Abcam) 1:10 000), phospho -Rb (Ser807/811) (D20B12) XP® Rabbit mAb (Cell Signalling #8516), Rb (4H1) Mouse mAb (Cell Signalling #9309)]. Membranes were washed in 1% w/v BSA TBS-T, secondary antibodies were incubated at room temperatures for 1 h [goat anti mouse IgG-HRP (Abcam) 1:5000; goat anti-rabbit HRP (Sigma–Aldrich) 1:5000]. Membranes were washed in TBS-T and imaged by chemiluminescence using SuperSignal West *Pico* PLUS Chemiluminescent Substrate (Thermo Fisher). Gels were imaged and quantified using an iBright system (InvitroGen).

### EdU labelling

Cells were grown in plates containing autoclaved coverslips and incubated with 10 µM EdU (Thermo Fisher Scientific) for 30 min. For synchronized cells post-quiescence, EdU was added at 15 h post-release and imaged at indicated timepoints. For restriction point analysis, EdU was added after addition of serum-free medium, and EdU-labelled cells were quantified at 24 h after release from quiescence. For all experiments, coverslips were removed and washed 3 × 3 min in 1 ml PBS. Coverslips were fixed with 0.2 ml 4% paraformaldehyde (PFA) in PBS for 15 min at room temperature. PFA was removed and coverslips washed 3 × 3 min with 1 ml PBS. EdU was fluorescently labelled using the Click-iT™ EdU Cell Proliferation Kit for Imaging (Thermo Fisher Scientific). Alexa Fluor 555 azide and Alexa Fluor 488 azide were used as indicated. After labelling and washing coverslips were mounted onto glass slides with VECTASHIELD® Antifade Mounting Media with DAPI (Vector Laboratories). Slides were imaged using a Zeiss Axiozoom fluorescence microscope, 100 nuclei were counted for each experiment, and all experiments were performed in triplicate. Coverslips were mounted in VectaShield with DAPI (VectaLabs).

### Flow cytometry

Cells were incubated with 10 µM EdU for 1 h and cells were harvested by trypsinization. Trypsin was quenched with DMEM, and cells were centrifuged at 500 × *g* for 5 min. Cells were washed three times in 1% BSA in PBS centrifuging for 5 min at 500 × *g* after each wash. Cells were permeabilized in 0.5% Triton X-100 in PBS for 20 min at 4°C. Cells were centrifuged and washed three times in 1% w/v BSA in PBS and centrifuged after each wash. Cells were labelled with 500 μl of EdU labelling cocktail (from Alexa Fluor 488 azide Click-iT™ EdU Cell Proliferation Kit) for 30 min at 4°C. Cells were washed three times in 0.1% Triton X-100 in PBS and centrifuged after each wash. Propidium Iodide was added (50 µg/ml). Cells were imaged using a Beckman Coulter CytoFLEX. Data analysis and figure creation were performed in the CytExpert software package (Beckman Coulter).

### Cell-free *in vitro* DNA replication assays

Cell-free DNA replication reactions were performed as described [14, 15]. To prepare synchronized G1 cytosolic extracts and replication-licensed G1 nuclei, NIH3T3 cells were synchronized by contact inhibition and released for 15 h (cytosolic extract) or 17.5 h (replication-licensed nuclei), respectively [14, 15]. To prepare S-phase cytosolic extracts, HeLa cells were synchronized by a double thymidine block. Initially, cells were incubated with 2 mM thymidine for 24 h, media were removed, cells were washed in PBS, and cells were released into fresh media for 8 h to progress through S-phase, and then incubated in 2 mM thymidine for 16 h. Cells were released into fresh medium for 1 h to allow replication to occur. S-phase cytosolic extracts were prepared by incubation of cells in ice cold hypotonic buffer [20 mM HEPES (pH 7.8), 15 mM potassium acetate, 0.5 mM MgCl_2_, 1 mM DTT], for 5 min. Cells were scrape-harvested and cells homogenized in a loose Dounce homogenizer (7 strokes for nuclei and 25 strokes for cytosolic extract preparation). Nuclei were isolated by centrifugation at 6000 × *g*, and cytosolic extracts were purified by 17 000 × *g* for 10 min. Nuclei and cytosolic extracts are flash-frozen in 10 and 30 μl aliquots in liquid nitrogen and stored in liquid nitrogen until use.

*In vitro* DNA replication assays utilize licensed G1 nuclei and either G1 or S-phase cytosolic extracts supplemented with 1:50 biotin-16-dUTP (Roche), 1:50 dilution 10 mg/ml creatine phosphokinase (Calbiochem), 1:50 1 M MgCl_2_, 10× premix (400 mM HEPES, pH 7.8, 70 mM MgCl_2_, 10 mM DTT, 400 mM phosphocreatine (CALBIOCHEM/MERCK), 30 mM ATP, 1 mM GTP, 1 mM CTP, 1 mM UTP, 1 mM dATP, 1 mM dGTP, 1 mM dCTP), and specified recombinant protein(s). Reactions were incubated for 30 min at 37°C. For microscopy analysis, nuclei were centrifuged through a 30% (v/v) sucrose solution onto poly-D-lysine coated coverslips. Samples were washed with PBS and antibody buffer (PBS, pH7.4, 0.1% v/v Triton X-100 and 0.02%w/v SDS), labelled for 30 min with streptavidin Alexa Fluor 555, washed again with antibody solution, and PBS, then mounted using VectaShield with DAPI. For immunoblotting, cell-free reactions were performed, and reactions were resuspended in an equal volume of 4× SDS–PAGE loading buffer, boiled, and analysed by SDS–PAGE and western blotting.

### RT-qPCR

Cell samples were harvested for RNA preparation by trypsinization, reaction were quenched in media, and cells were collected by centrifugation. RNA samples were isolated using the PureLink™ RNA Mini Kit (Invitrogen) and stored at −80°C. RT-qPCR was performed using Taqman primers from Invitrogen: cyclin D1 (Mm00432359_m1), cyclin E1 (Mm01266311_m1), cyclin E2 (Mm00438077_m1), cyclin A2 (Mm00438063_m1) and GAPDH (Mm99999915_g1), CDKn2b (Mm00483241_m1), CCND1 (Mm00432359_m1), CDKN1A (Mm04207341_m1), CDK2Nc (Mm00483243_m1)

Gene Symbol: Cdkn2b (Mm07295536_m1). RT-qPCR was performed using the Luna® Universal Probe One-Step RT-qPCR Kit (New England Biolabs), on a BioRad C1000 CFX96 touch screen thermal cycler. DDCt values were calculated and displayed as relative quantitation (RQ).

### DNA combing

Silanized coverslips were prepared using the method described in Schwob *et al.* (2008). DNA was isolated from 1.2 × 10^7^ cells and purified within agarose plugs using the BIORAD CHEF genomic DNA plug set. Plugs were incubated overnight in proteinase K at 50°C and washed 4 times in TNE50 (incorporating 1 mM PMSF on the third wash). The day before combing, plugs were incubated for 30 min in 10 mM MES-ethylenediaminetetraacetic acid (EDTA), pH 5.7, then 30 min at 65°C in 10 MES, 1 mM EDTA, pH 5.7, then plugs were incubated overnight at 42°C in MES-EDTA containing β-agarase. Silanized coverslips were placed in DNA solution for 10 min and withdrawn at a constant rate of 18 mm/min. Coverslips were incubated at 50°C for 1 h, then prepared for imaging by immunofluorescence.

### Antibody labelling of IdU, CldU, and ssDNA

After DNA combing onto coverslips, all wash steps were performed in Coplin jars. Coverslips were incubated at 60°C for 1 h. DNA was denatured in 0.5 M NaOH for 30 min at room temperature. Slides were washed 3 × 3 min in washing buffer (0.1% v/v Tween 20, PBS, pH 7.4), transferred to a humidity chamber, and blocked in 200 μl blocking buffer (3% BSA, 0.1% v/v Tween 20, PBS, pH 7.4) at 37°C for 1 h. Blocking buffer was removed, and 50 μl of primary antibody solution 1 was added (Table 3). Primary antibody solutions were incubated overnight at 4°C.

**Table 3.** Antibodies used for DNA combing

Solution	Antibody	Species raised	Concentration	Supplier
Primary antibody solution 1	IdU	Mouse	1/20	BD Biosciences 347580
	CldU	Rat	1/125	Serotec OBT0030
Primary antibody solution 2	ssDNA	Mouse	1/50	Merck Millipore MAB3034
Secondary antibody solution 1	Anti-Rat IgG Alexa Fluor 488	Chicken	1/50	Thermo Fisher Scientific A-21470
	Anti-Chicken IgY Alexa Fluor 488	Goat	1/50	Thermo Fisher Scientific A-11039
	Anti-Mouse IgG Alexa Fluor 633	Goat	1/50	Thermo Fisher Scientific A-21050
Secondary antibody solution 2	Anti-Mouse IgG Alexa Fluor 568	Rabbit	1/50	Thermo Fisher Scientific A-11061
	Anti-Rabbit IgG Alexa Fluor 568	Goat	1/50	Thermo Fisher Scientific A-11036
Primary antibody solution 3	Digoxigenin	Mouse	1/50	Abcam (AB420)
Secondary antibody solution 3	Anti-Mouse IgG Alexa Fluor 633	Goat	1/50	Thermo Fisher Scientific A-21050

Slides were washed 3 × 3 min in washing buffer, transferred to a humidity chamber, and 50 μl of secondary antibody solution 1 (Table 3) was added and incubated at 37°C for 30 min. Slides were washed 3 × 3 min in washing buffer, transferred to a humidity chamber, and 50 μl of primary antibody solution 2 was incubated for 2 h at room temperature. Slides were washed 3 × 3 min in washing buffer, and 50 μl of secondary antibody solution 2 was added and incubated for 30 min at 37°C. Slides were washed 3 × 3 min in washing buffer, and slides were mounted on a 22 × 22 mm square coverslip with ProLong™ Gold Antifade Mount (Thermo Fisher Scientific).

### Statistical analysis

For analysis of G1 length and cell cycle length for WT versus CIZ1 KO cells. Data were assessed for normality using D’Agostino & Pearson and Anderson-Darling test at 95% confidence limits and were found to be normally distributed. Data were assessed using a two-tailed unpaired *t*-test using GraphPad Prism software. RT-qPCR was analysed using GraphPad Prism using a two-way analysis of variance (ANOVA) followed by pairwise Fisher’s LSD comparison tests. Cyclin A chromatin levels were found to be non-parametric and analysed using Kruskal–Wallis’s test followed by Tukey’s multiple comparisons test using GraphPad Prism software. Where ** = *P *< .01, *** =*P *< .001. DNA combing data were assessed for normality and were not found to be normally distributed (Fig. 8G). Data were analysed using non-parametric ANOVA analysis using the Kruskal–Wallis’s test followed by Dunn’s multiple comparisons test using GraphPad Prism software. Where *=*P *< .05, ** = *P < *.01, *** =*P *< .001, and **** = *P *< .0001. Cell-free DNA replication analyses found data were normally distributed and analysed by two-way ANOVA followed by pairwise post hoc tests using Tukey’s Honestly Significant Difference multiple comparisons. Where *** =*P *< .001 and **** = *P *< .0001. Data were analysed in Fig. 7 after normality testing using GraphPad Prism. Data shown were parametric for A, B, and D, and statistical testing was performed using one-way ANOVA with Tukey’s multiple comparisons test where *=*P *< .05, ** = *P *< .01, and **** = *P *< .0001. Data in Supplementary Fig. S7C were non-parametric and analysed using one-way ANOVA using Krustal–Wallis multiple comparison test, where **** = *P *< .0001.

## Results

### CIZ1 ablation reduces G1 and cell cycle length in post-quiescent cells

**Figure 1.**
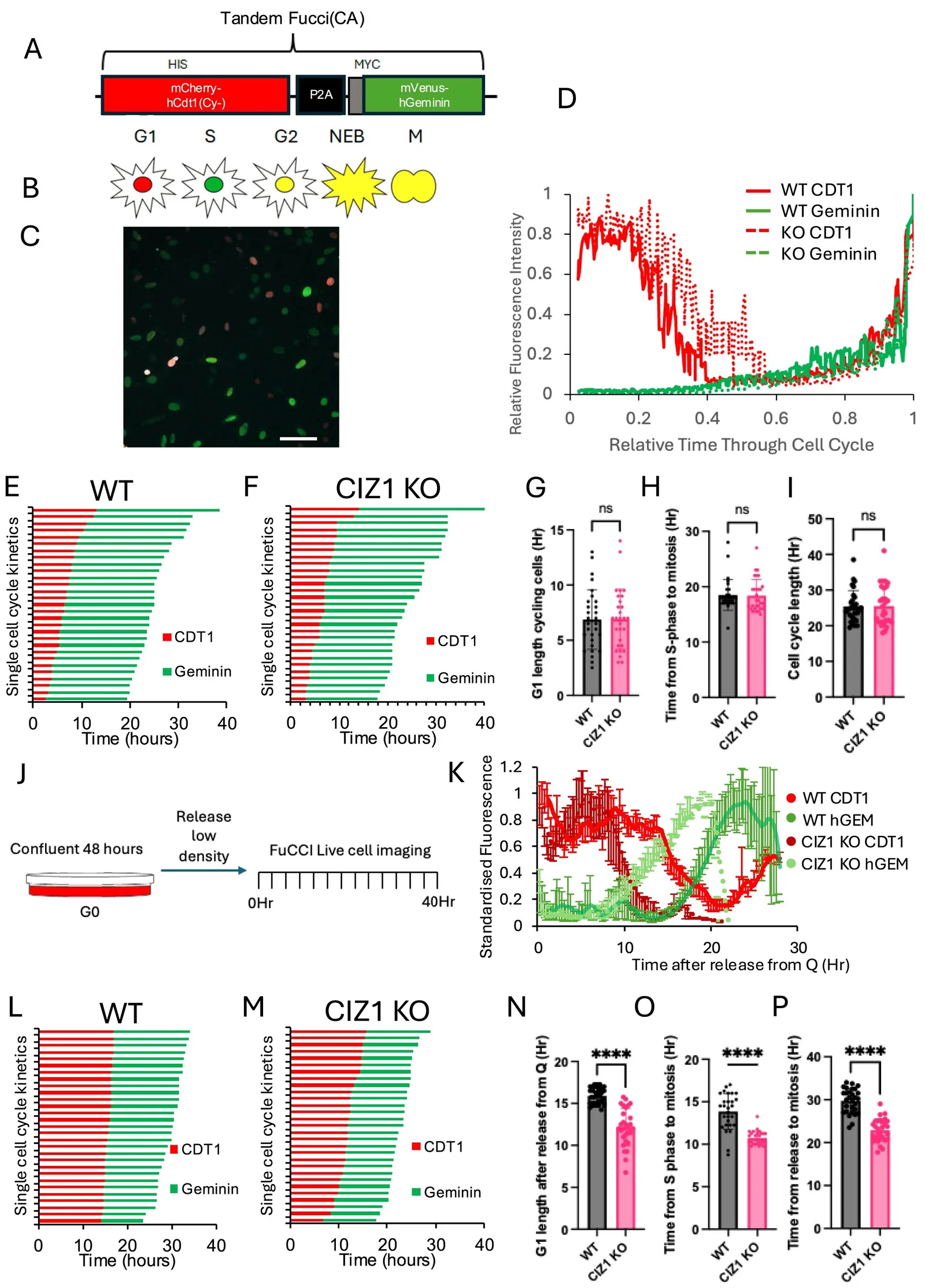
CIZ1 ablation reduces G1 length in post-quiescent fibroblasts. (**A**) Cartoon of the bicistronic Tandem Fucci(CA) construct, showing mVenus-Geminin and mCherry hCdt1 (Cy-) fused with the porcine teschovirus-1 2A peptide sequence. (**B**) Colour scheme for each cell cycle phase for Fucci(CA). (**C**) Representative fluorescence microscope image of Fucci(CA) murine fibroblasts. White scale bar = 50 μm. (**D**) Interpolated fluorescence profile for WT and CIZ1 KO fibroblasts in cyclin cells. Zero hours refers to mitosis, allowing alignment of cell cycle phases. Data show mean fluorescence for mCherry-Cdt1 (red) and mVenus-Geminin (Green) for WT (solid lines) and CIZ1 KO cells (dashed lines). (**E**) Plot of WT and (**F**) KO cells, showing mCherry-CDT1 and for mVenus-geminin fluorescence after mitosis showing G1 length (red) and S-M length (green). (**G**) Plot of median G1 length, showing single-cell median degradation of mCherry-Cdt1 and mVenus-Geminin accumulation. Bar charts show mean value and standard deviation. Statistical significance was determined using two-tailed unpaired *t*-test. *n* = 30. (**H**) Plot of time from S-phase to M phase (stable geminin expression, green bars from panels E and F). Individual cell data shown with bar charts showing mean and standard deviation, *n* = 30. Statistical significance was determined using two-tailed unpaired t-test. (**I**) Plot of cell cycle length, showing single-cell data for WT and CIZ1 KO cells, respectively, from mitosis to mitosis. Individual cell data shown with bar charts showing mean and standard deviation, *n* = 30. Statistical significance was determined using two-tailed unpaired *t*-test. (**J**) Experimental overview of cell sychronization in G0 by contact inhibition and post-quiescence Fucci(CA) cell cycle imaging procedure. (**K**) Average fluorescence signal for mCherry hCdt1 and mVenus geminin after release from quiescence. WT cells (Solid line) and CIZ1 KO (dashed lines), showing G1 length (red, mCherry-Cdt1) and S-M (green, mVenus-geminin). Data points show mean fluorescence with standard deviation. (**L**) Plot of representative cells for WT and (M) KO cells, showing timepoint of median fluorescence decrease for mCherry-CDT1 and increase for mVenus-geminin. (**N**) Plot of median G1 length from panels (L, M). Individual cell data shown with bar charts showing mean G1 length and standard deviation. Statistical significance was determined using two-tailed unpaired *t*-test, (*n* = 30, *P *< .0001). (**O**) Plot showing cell cycle length S phase to mitosis. Individual cell data shown with bar charts showing mean and standard deviation. (**P**) Plot showing cell cycle length to mitosis. Individual cell data shown with bar charts showing mean and standard deviation Statistical significance was determined using two-tailed unpaired *t*-test (*n* = 30, *P *< .0001).

To investigate the potential role of CIZ1 in regulation of cell cycle kinetics, *CIZ1^−/−^* cells were produced using CRISPR-Cas9 (CIZ1 KO) in murine NIH3T3 fibroblasts (Supplementary Fig. S1A and B). CIZ1 KO clones were identified by western blotting (Supplementary Fig. S1C), and frame shifts confirmed using nested PCR and DNA sequencing to confirm *CIZ1* knock out (Supplementary Fig. S1D). In verified CIZ1 KO cells lines, the tandem Fucci(CA) bicistronic cell cycle biosensor [39] was stably integrated using PiggyBac integration [43] to facilitate cell cycle analyses. The Fucci(CA) system encodes truncated versions of mCherry-CDT1 and mVenus-Geminin that are regulated by CUL4 and APC/C-CDH1 ubiquitin ligases respectively (Fig. 1A).

Degradation of CDT1 enables visualization of the G1/S transition, as CDT1 is degraded by the S-phase specific E3 ligase SCF-CUL4. The accumulation of mVenus-Geminin from S-phase to mitosis following inactivation of APC/C-CDH1 provides a second marker of the G1/S transition (Fig. 1B and C). Analysis of exponentially dividing WT parental cells and CIZ1 KO cells by Fucci(CA) live cell imaging enabled precise quantification of G1 length and cell cycle length. Interpolation of a single cell cycle, where cells are standardized by mitosis (time point 0) and followed for 1 complete cell cycle at mitosis showed similar degradation kinetics for mCherry-Cdt1 and mVenus-Geminin accumulation (Fig. 1D). Determination of G1 length, S-M phase length and total cell cycle length revealed no significant differences (Fig. 1E–I). Monitoring G1 length (Fig. 1G) identified no difference in G1 length (6.8 ± 2.7 and 6.9 ± 2.7 h, respectively) (Fig. 1E–G), no difference in S-M length (Fig. 1H) and no significant difference in cell cycle length (mitosis to mitosis) in WT parental cells and CIZ1 KO cells (Fig. 1I). Similarly, asynchronous cells showed similar cell cycle profiles by flow cytometry (Supplementary Fig. S2A). Collectively, the data show that CIZ1 is not essential for regulation of cell cycle length in exponentially dividing murine fibroblasts, consistent with its non-essential developmental role [8, 17–19].

CIZ1 ablation causes epigenetic instability after successive quiescence-proliferation cycles, which underpins cellular transformation in murine fibroblasts [21]. We therefore reasoned that CIZ1 may regulate cell cycle re-entry kinetics after quiescence. WT and CIZ1 KO Fucci(CA) cells were synchronized in G0 by contact inhibition and released into G1 phase allowing post-quiescence cell cycle kinetics to be observed (Fig. 1J and Supplementary Fig. S3B and C). Monitoring interpolated mCherry-Cdt1 and mVenus-Geminin expression levels identified that CIZ1 KO cells reduce CDT1 levels and increase mVenus-Geminin expression earlier than WT cells, consistent with reduction in G1 length (Fig. 1K and L).

Monitoring the timepoint where CDT1 is degraded (determined as time point where <50% maximum signal) in post-quiescent cells identified that CIZ1 KO significantly reduces the mean length of G1 phase relative to WT cells (Fig. 1L–N) (WT, 15.9 ± 0.9 h versus KO, 12.14 ± 2.2 h, *P *< .0001; Fig. 1N). In addition, CIZ1 KO cells also showed a significant reduction in S-phase to M phase length (Fig. 1O), resulting in a significant reduction in cell cycle length from quiescence to mitosis (WT 29.7 ± 2.8 h versus 22.87 ± 2.7 h for CIZ1 KO, *P *< .0001; Fig. 1P, Supplementary Fig. S3). These data suggest that CIZ1 contributes to mechanisms that establish both G1 and cell cycle length in post-quiescent cells.

### CIZ1 ablation increases intracellular CDK activity, reducing G1 length in post-quiescent cells

**Figure 2.**
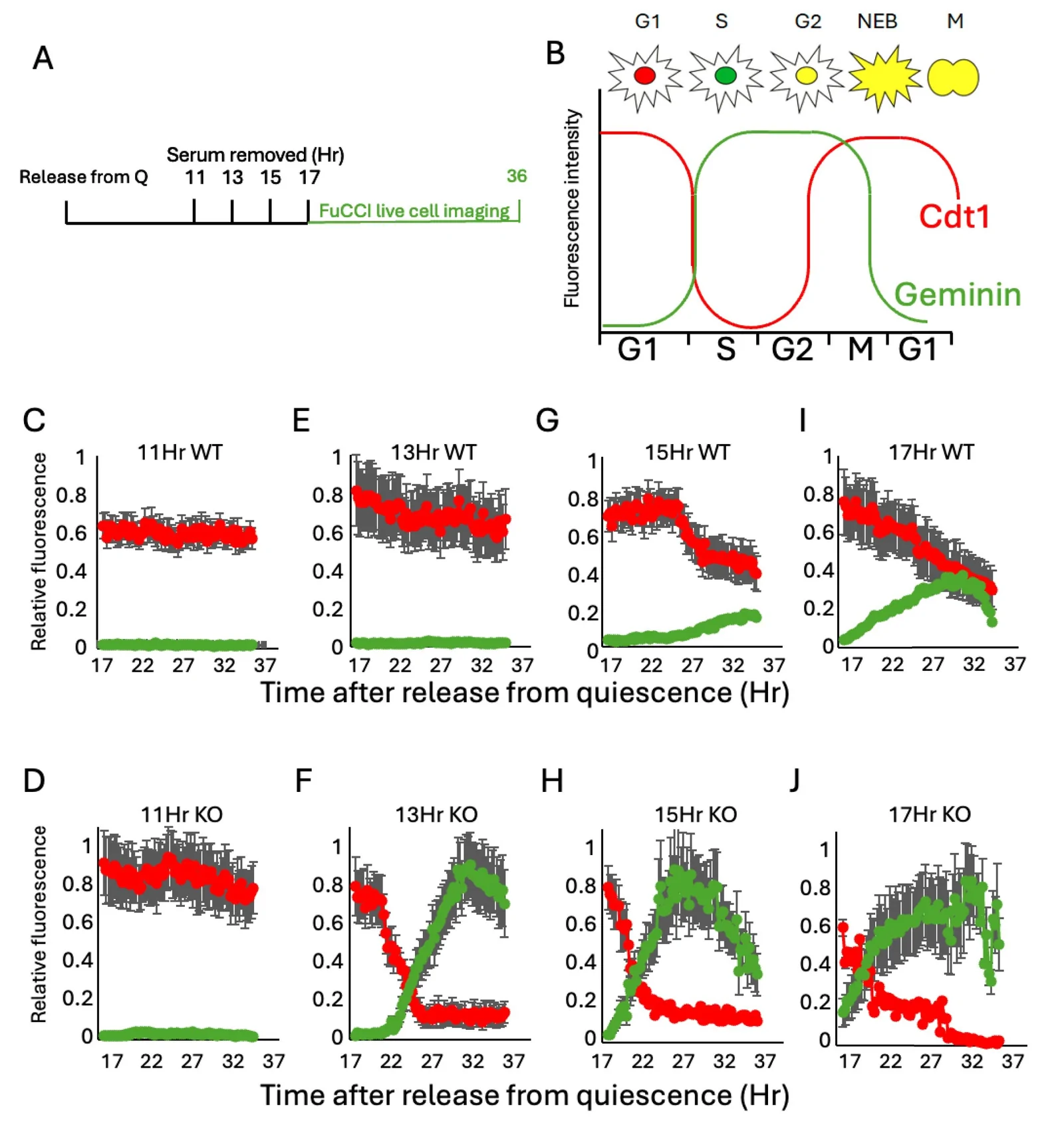
CIZ1 KO cells bypass restriction point before WT post-quiescent cells. (**A**) Experimental overview. Cells are synchronized by contact inhibition and released by dilution in fresh media. Serum is removed at indicated time points and live cell imaging performed. (**B**) Cartoon of Fucci(CA) fluorescence by cell cycle stage and relative expression of mCherry-Cdt1 (red) and mVenus-Geminin (green) shown by cell cycle stage. (**C**) WT Fucci(CA), (**D**) CIZ1 KO Fucci(CA) cells with serum removed 11 h after quiescence. Cells remain in G1 phase. (**E**) WT Fucci(CA), (**F**) CIZ1 KO Fucci(CA) cells with serum removed 13 h after quiescence. WT cells remain in G1 phase, but CIZ1 KO cells exit G1 phase. (**G**) WT Fucci(CA), and (**H**) CIZ1 KO Fucci(CA) cells with serum removed 15 h after quiescence. WT cells enter S-phase with a delay relative to CIZ1 KO cells. (**I**) WT Fucci(CA), and (**J**) CIZ1 KO Fucci(CA) cells with serum removed 17 h after quiescence. Both WT and CIZ1 KO cells enter S-phase. All data show the mean fluorescence standardized to maximum detected signal for Cdt1-mScarlet and Geminin-mVenus per condition and showing standard deviation, *n* = 20.

G1 length plays a key role in genome stability and cell fate decisions. G1 length is regulated by intracellular CDK activity [44] with increasing CDK activity required to bypass the restriction point and initiate DNA replication. To investigate the effect of CIZ1 KO on restriction point bypass, Fucci(CA) live cell imaging was performed on post-quiescent cells re-entering the cell cycle with serum removed at specified time points pre- and post-restriction point, with mitogen independent cell cycle progression identifying restriction point timing (Fig. 2).

WT and CIZ1 KO Fucci(CA) cells were synchronized in G0 by contact inhibition and released from quiescence into complete medium. At indicated timepoints serum was removed and cells cultured in serum free medium to identify restriction point timing monitoring cell cycle progression from 17 to 36 h post-quiescence (Fig. 2). The fluorescence intensity for CDT1-mCherry and Geminin-mVenus were determined for a population of cells, and mean fluorescence signal presented (Fig. 2A and B). As the G1/S transition is marked by activation of S-phase ubiquitin ligase SCF-CUL4 and inactivation of APC/C-CDH1 there is a loss of CDT1-mCherry signal and increase in mVenus-geminin signal at the G1/S transition.

Live cell imaging of WT and CIZ1 KO Fucci(CA) cells at 11–17 h serum removal showed distinct effects on mCherry-Cdt1 degradation, representing G1 exit and restriction point bypass in CIZ1 KO cells relative to WT cells. Removal of serum at 11 h blocks cell cycle progression for both WT and CIZ1 KO cells, as indicated with stable mCherry-CDT1 accumulation (Fig. 2C and D). However, there is a differential response in WT and CIZ1 KO cells in cells where serum is removed 13 h after release from quiescence (Fig. 2E and F). WT cells could not re-enter the cell cycle after serum remove 13 h after release, whereas CIZ1 KO cells were able to bypass restriction point and exit G1 phase (Fig. 2E and F). Similarly, CIZ1 KO cells are no longer mitogen dependent for G1 exit where serum is removed 15 h after release, but at this time point WT cells can also exit G1 phase, albeit with a longer G1 length (Fig. 2G and H). Both WT and CIZ1 KO cells exit G1 phase where serum is removed 17 h after release from quiescence (Fig. 2I and J). These data show that CIZ1 KO cells bypass restriction point and become serum independent earlier than WT cells, identifying an earlier restriction point bypass in post-quiescent CIZ1 KO cells.

**Figure 3.**
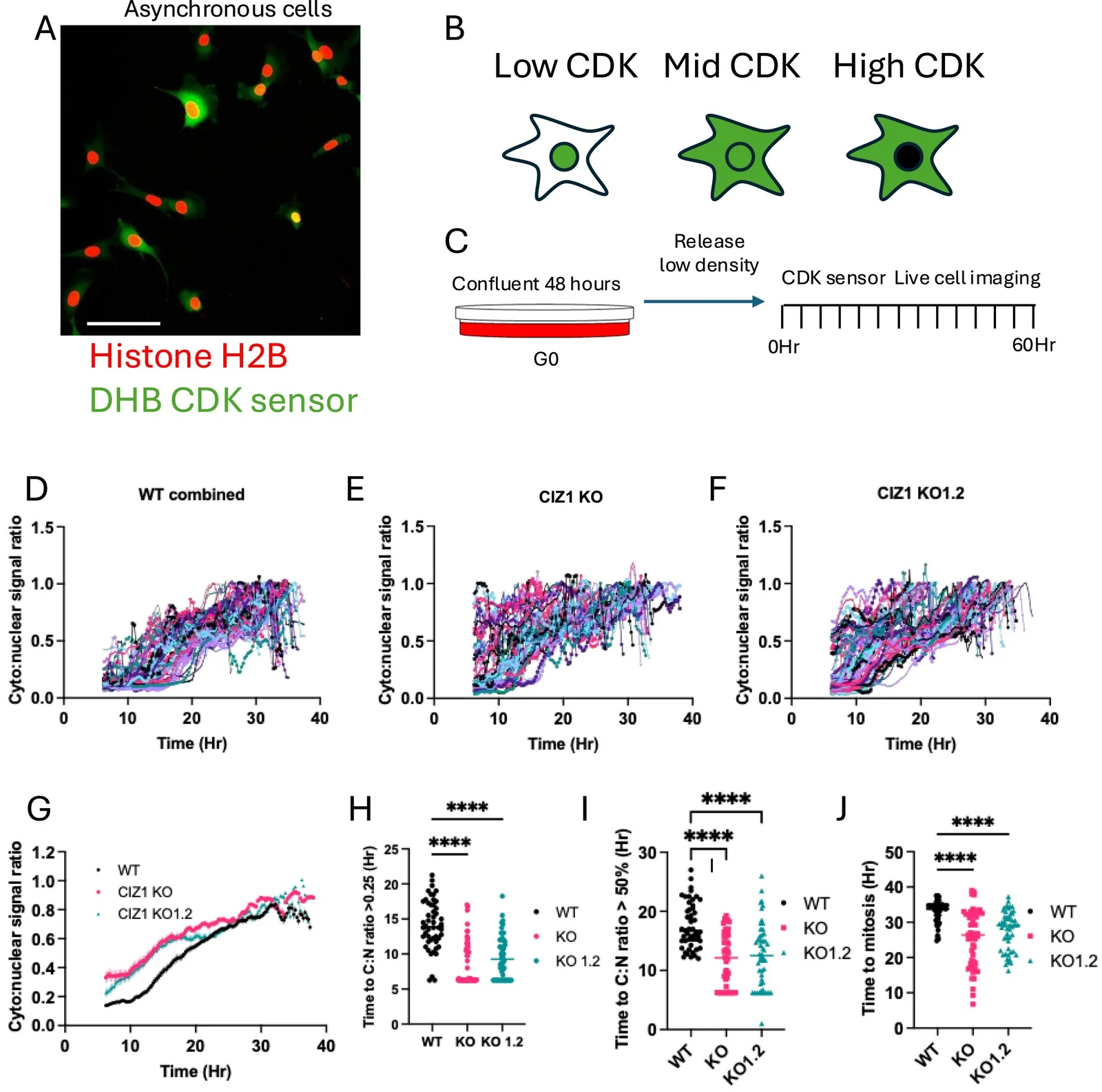
Post-quiescent CIZ1 KO cells have increased intracellular CDK activity resulting in reduced cell cycle length. (**A**) Representative image of mNeon green-DHB1 and mScarlet-Histone 2B in asynchronous WT 3T3 cells. White scale bar = 50 μm. (**B**) Cartoon image of the localization of mNeon green-DHB1 in low, mid and high CDK environments. DHB localizes to the nucleus at low CDK activity and is cytoplasmic at high CDK activity. (**C**) Overview of experimental procedure. 3T3 cells were released from contact inhibition and live cell imaging performed post release. Single cell CDK from 3 biological replicates and 50 nuclei imaged in total for WT (**D**), CIZ1 KO (**E**) and CIZ1 KO1.2 (**F**). Data show ratio of mNeon green fluorescence in the cytoplasm relative to nucleus and normalized to a value of 1 for maximum fluorescence that typically peaks just prior to mitosis. (**G**) Mean fluorescence intensity was plotted for WT (black), CIZ1 KO (red) and CIZ1 KO1.2 (green) using data from 50 cells for each cell line from panels (D–F). (**H**) To determine the intracellular CDK activity, the time at which the fluorescence intensity reached >0.25 and plot for WT, CIZ1 KO, and CIZ1 KO1.2. Statistical testing was performed using non-parametric one-way ANOVA analysis, using Krustal Wallis test and Dunn’s multiple comparisons test. Each data point represents 1 cell, *n* = 50. ****, where *P *< .0001. I) As for H, using >0.5 as a threshold. (**J**) Plot showing the time to mitosis for 50 WT, CIZ1 KO, and CIZ1 KO1.2 cells. Statistical testing was performed using non-parametric one-way ANOVA analysis, using Krustal Wallis test and Dunn’s multiple comparisons test. Each data point represents 1 cell, *n* = 50. **** = *P *< .0001.

This striking difference in G1 length and earlier restriction point bypass suggests that CIZ1 KO cells may have altered intracellular CDK activity relative to WT cells. As CIZ1 has been implicated in regulation of cyclin-CDK activity and CDK activity sensor at the G1/S transition [14, 15], these data suggest that CDK networks may be dysregulated in the absence of CIZ1. To investigate the effects of CIZ1 on intracellular CDK activity, the intracellular CDK activity reporter DHB was used [22, 25]. The DHB CDK sensor was fused to mNeon green (DHB-mNeon Green) and H2B-mScarlet was also cloned into a biscitronic expression system with a self-cleaving P2A peptide to detect intracellular CDK activity. Intracellular CDK activity levels can be determined by visualization of the cytoplasmic and nuclear DHB fluorescence signal ratio [22, 25]. A stable construct expressing this reporter was produced for WT, CIZ1 KO and a second CIZ1 KO cell line (CIZ1 KO1.2) to facilitate analysis (Fig. 3A–C). To determine the intracellular CDK activity in post-quiescent cells specifically, cells were synchonized in G0, released and the intracellular CDK activity in cells re-entering the cell cycle prior to the first mitosis were determined. All cell lines showed a heterogenous range of CDK activity in post-quiescent cells but both CIZ1 KO cells lines showed an increase in CDK activity during early timepoints relative to WT cells (Fig. 3D–F). To determine the CDK activity, the mean cytoplasm:nuclear fluorescence signal was determined in WT, CIZ1 KO, and CIZ1 KO1.2 cells, which showed higher CDK activity in the both CIZ1 KO mutants relative to WT cells at early time points before 20 h post-release from quiescence (Fig. 3G). To quantify and evaluate these differences, two different cytoplasm to nuclear fluorescence ratios were used at 0.25 and 0.5 and the timepoint where these values were reached (Fig. 3H and I).

Both CIZ1 KO cell lines have higher CDK activity earlier in the cell cycle and reach 25% (Fig. 3H) and 50% (Fig. 3I) of the maximum CDK activity, significantly earlier than WT cells, demonstrating a high intrinsic CDK activity in CIZ1 KO cells. The increased CDK activity results in CIZ1 KO cells having a significantly reduced cell cycle length relative to WT cells (Fig. 3J). These data corroborate those found Fucci(CA) system (Fig. 1) and demonstrate that post-quiescent CIZ1 KO cells have increased intracellular CDK activity relative to WT parental cell lines.

### CIZ1 KO cells have reduced G1 length and increased G1/S cyclin expression

The observed increase in intracellular CDK activity, reduction in G1 length and earlier restriction point timing in post quiescent CIZ1 KO cells, suggests dysregulation of the CDK network in CIZ1 KO cells. We hypothesized that there may be altered G1 cyclin or CDK inhibitor protein expression resulting in increased CDK activity in CIZ1 KO cells in G1 phase. Initially, the levels of cyclin D, cyclin E and cyclin A mRNA levels were determined in asynchronous cells (Supplementary Fig. S4). *CCND1, CCNE1, CCNE2*, and *CCNA2* all show significant increases in mRNA levels relative to WT cells. As CDK inhibitor proteins can also influence cyclin CDK activity, the levels of p21, p27, p57, and INK4 transcript levels were assessed. There was an increase in p21 expression, but expression of p27 was reduced and no significant changes in INK4 or p57 transcript levels were found. The data suggest that CIZ1 KO cells overexpress G1 and S phase cyclin genes that could underpin enhanced CDK activity.

**Figure 4.**
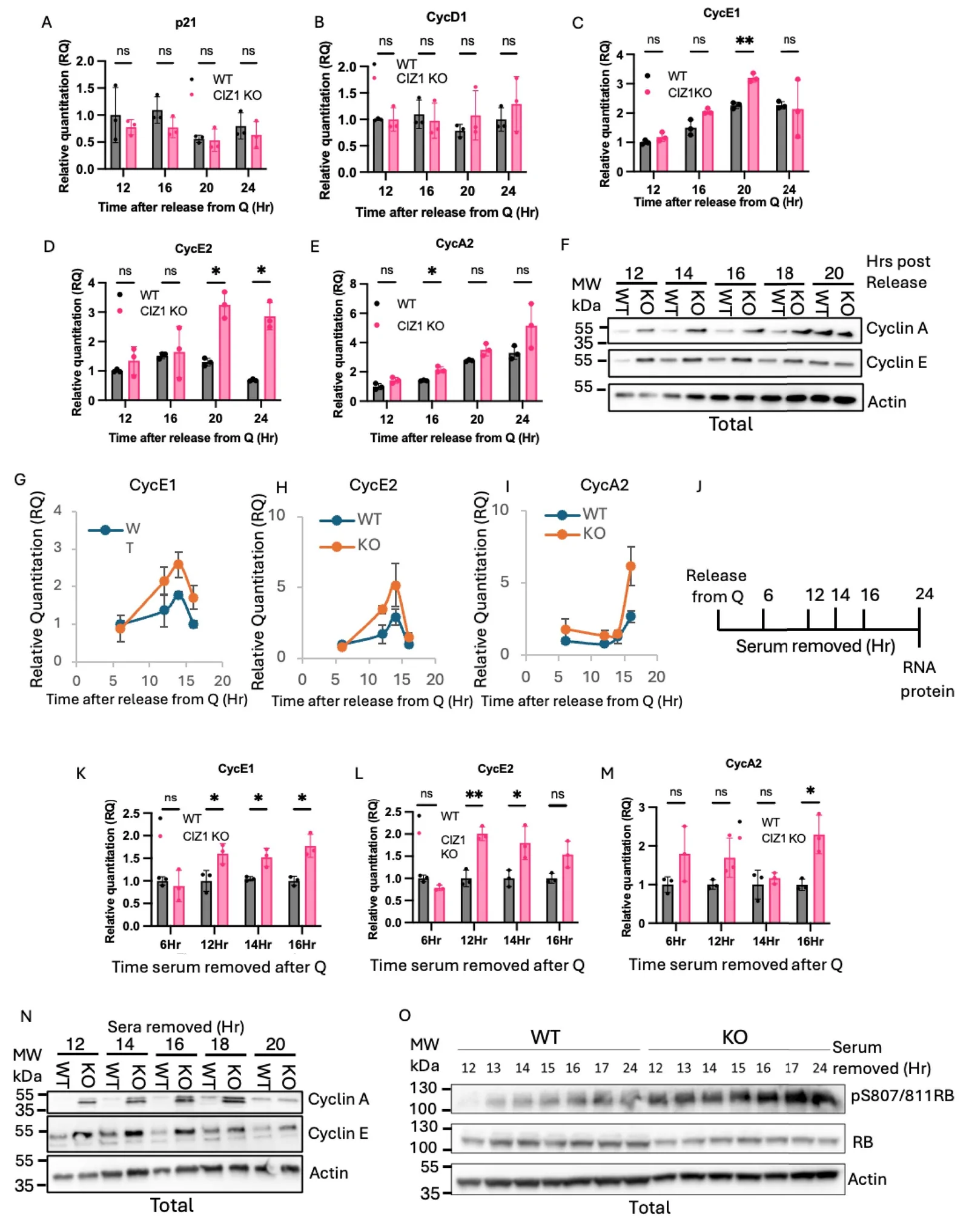
CIZ1 ablation increases cyclin E1, cyclin E2 and cyclin A2 expression in post-quiescent cells reduces G1 length through early R bypass. (**A-E**) RT-qPCR monitoring expression of *p21* (**A**), *cyclin D1* (**B**), *Cyclin E1* (**C**), *cyclin E2* (**D**), and *cyclin A2* (**E**) in WT and CIZ1 KO cells in synchronous post-quiescent cells. Data show mean ± standard deviation, where *n* = 3. Statistical analysis was performed using two-way ANOVA Fisher’s LSD comparisons test. * - *P *< .05 and ** *P *< .01. F) Western blot of whole cell lysate from cells released from quiescence at specified timepoints showing cyclin E, cyclin A and actin. (G–J) mRNA levels are shown relative to actin for WT and CIZ1 KO cells at indicated time points for *cyclin E1* (**G**) *cyclin E2* (**H**), and *cyclin A2* (**I**). (J) Experimental overview. Cells were released from quiescence and serum removed at time indicated time points. mRNA levels were quantified 20 h post release. (K-M) Bar chart and statistical analysis of *Cyclin E1* (**I**), *cyclin E2* (**J**), and *cyclin A2* (**K**) mRNA levels where serum was removed at indicated timepoints. Data show mean ± standard deviation, where *n* = 3. Statistical analysis was performed using two-way ANOVA Fisher’s LSD comparisons test. * - *P *< .05 and ** *P *< .01. (N) Western blot of whole cell lysate from cells released from quiescence and serum removed at specified timepoints. Protein was harvested at 24 h post release from quiescence. Blots show cyclin E, cyclin A and actin. (O) Western blot of whole cell lysates showing RB and phospho-RB levels in WT and CIZ1 KO cells. Time points indicate where serum was removed, and cell cultured in serum free media. Cells were harvested 24 h after release from quiescence.

Next the p21 and cyclin mRNA levels were determined in post-quiescent cells. The expression of p21 and cyclin D1 were unaffected (Fig. 4A and B) but E2F1-3 regulated cyclins, cyclin E1, E2, and A2 were all expressed at higher levels in CIZ1 KO cells relative to WT cells. Cyclin E1 was significantly overexpressed 20 h after release from quiescence, cyclin E2 at 20 and 24-h and cyclin A2 16 h post-quiescence (Fig. 4C–E). Analysis of the levels of cyclin E and cyclin A in quiescence showed similar levels of each cyclin in WT and CIZ1 KO cells, suggesting that differences in expression occur in G1 phase (Supplementary Fig. S5). Overall, these data suggest that CIZ1 KO cells have increased CDK activity relative to WT cells. Analysis of cyclin E and cyclin A expression in post-quiescent cells showed an increase in cyclin E and A levels between 12–18 h after release before plateauing at 20 h (Fig. 4F and Supplementary Fig. S4L and M). However, cyclin A protein levels are markedly increased in CIZ1 KO cells at all time points (Fig. 4F and Supplementary Fig. S4M). As cyclin A2 is polyubiquitylated by APC/C-CDH1 in G1 phase [45], this is consistent with CIZ1 KO cells exiting G1 phase earlier than WT cells.

To determine restriction point timing, cells were released from quiescence and serum removed as indicated (Fig. 4J). Cyclin E1 and E2 expression was significantly increased after removal of serum at 12 and 14 h after release from quiescence (qPCR performed at 24 h after release) and cyclin A2 when serum was removed at 16 h (Fig. 4K–M). Evaluation of cyclin E and A protein expression in cells after removal of serum showed an increase in cyclin E and cyclin A expression between 12 and 20 h (Fig. 4N). The increased expression of cyclin E and cyclin A correlates with increased RB phosphorylation at earlier time points and at higher levels relative to WT cells (Fig. 4O). These data suggest that expression of cyclins E1, E2, and A2 are increased in CIZ1 KO cells promoting earlier restriction point bypass.

Cyclin E and cyclin A perform distinct roles in the G1/S transition. Cyclin E is required for restriction point bypass and cell cycle re-entry following quiescence [46, 47] and cyclin A is functionally redundant in fibroblasts [48]. To investigate the role of cyclin E and cyclin A in a CIZ1 KO context, siRNA mediated depletion was used. Depletion of either cyclin E (*CCNE1/CCNE2*) or cyclin A (*CCNA2*) prevents S-phase entry by 24 h post release and significantly reduced mitosis within 50 h of release from quiescence (Supplementary Fig. S6F). Depletion of either cyclin E or cyclin A significantly reduced intracellular CDK activity in both WT and CIZ1 KO cells, with CDK sensor live cell showing a plateauing at around 0.6 nucleus to cytoplasmic fluorescence ratio (Supplementary Fig. S6G–L). The averaged data show that CIZ1 KO cells have increased CDK activity relative to WT cells, and depletion of either cyclin E1/E2 or cyclin A2 results in low intracellular CDK activity that is insufficient to complete the cell cycle.

There are significant differences in intracellular CDK activity at 15, 20, and 30 h after release between control siRNA treated cells and cyclin E or cyclin A depleted cells, but no significant differences in CDK activity levels between WT and CIZ1 KO cells (Supplementary Fig. S7). The data show that cyclin E or cyclin A depletion results in low CDK activities that are insufficient to drive cells into S-phase and compete the cell cycle in either WT or CIZ1 KO cells. This approach was unable to disentangle the specific roles of cyclin E and cyclin A and their activities at the G1/S transition, requiring alternative approaches to investigate the role of cyclin E and A at the G1/S transition.

### CIZ1 KO cells are deficient in cyclin A–CDK2 chromatin localization

**Figure 5.**
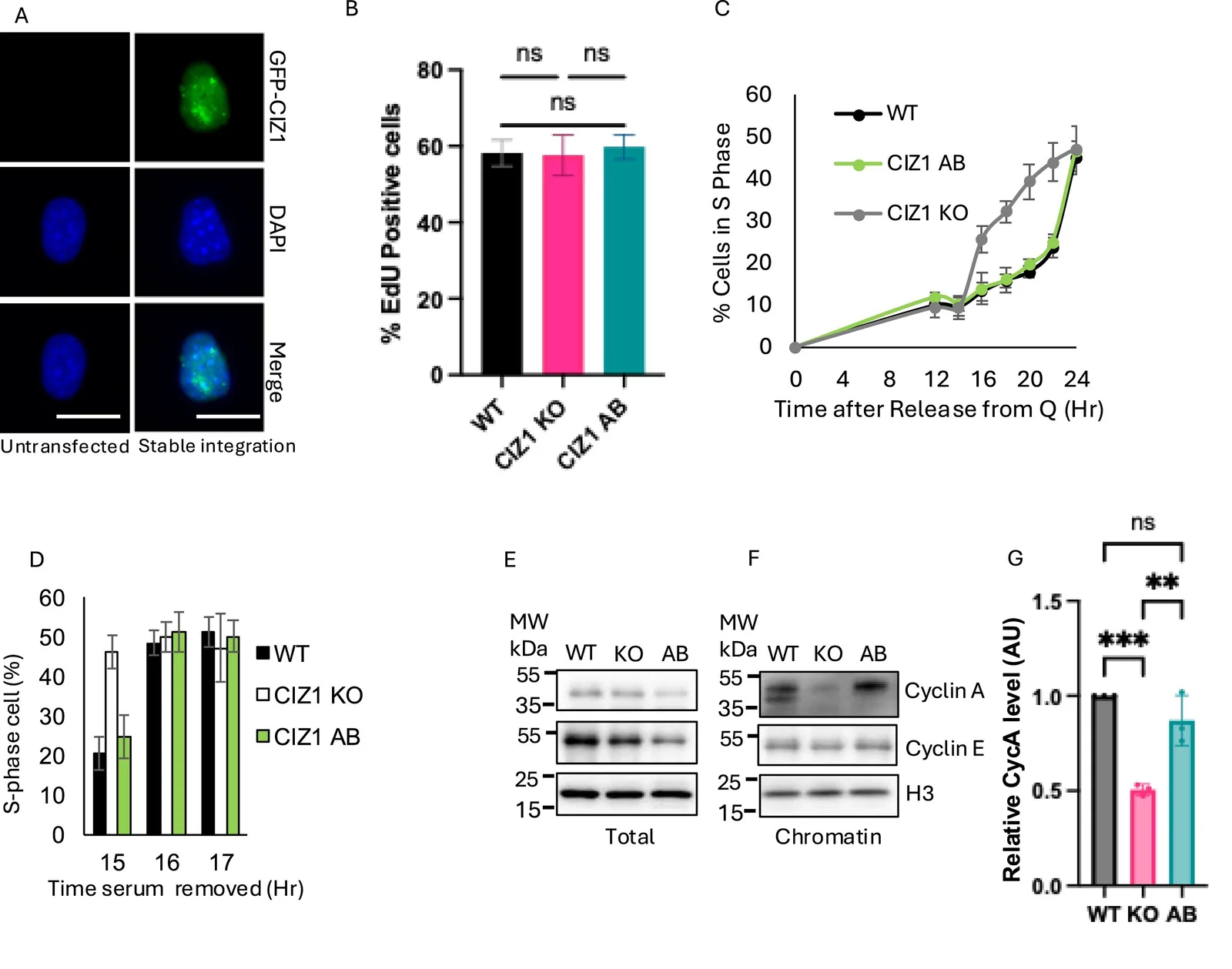
Post-quiescent CIZ1 KO fibroblasts have mislocalized cyclin dependent kinase localization. (**A**) Fluorescence microscope of GFP-CIZ1 expressing CIZ1 KO fibroblasts, CIZ1 add back (CIZ1 AB) cells. (**B**) Cells were pulse labelled with EdU for 30 min and percentage of S-phase WT, CIZ1 KO and CIZ1 AB cells shown. (**C**) Synchronized WT, CIZ1 KO and CIZ1 AB cells were pulse labelled with EdU, percentage of EdU labelled cells are shown from 0 to 24 h after release. (**D**) Post-quiescent cells were incubated with EdU after removal of serum at indicated time points and EdU positive cells counted at 24 h post-quiescence. (**E**) Western blot of synchronous cells 20 h after release from quiescence showing WCE showing cyclin A, cyclin E, and Actin. (**F**) Western blot of chromatin fraction showing cyclin A, cyclin E and Histone 2B (**G**) Quantitation of cyclin A on chromatin, bars show mean abundance ± standard deviation, where *n* = 3. Statistical analysis was performed using 1-way ANOVA with Tukey’s multiple comparisons test using GraphPad Prism software. Where ** = *P *< .01, *** =*P *< .001.

The observed differences in G1 length in post-quiescent WT and CIZ1 KO cells suggest that CIZ1 contributes to mechanisms that regulate G1 length in the first cell cycle following quiescence or G0. To assess if ectopic expression of CIZ1 can reverse the observed reduction in G1 length, GFP-CIZ1 was stably integrated into the chromosome of CIZ1 KO cells (CIZ1 add back, CIZ1 AB) (Fig. 5A).

Analysis of WT, CIZ1 KO or CIZ1 AB asynchronous cells following incubation with EdU showed no significant differences in the numbers of cells in S-phase (Fig. 5B). However, CIZ1 KO cells enter the S-phase several hours before WT, consistent with Fucci(CA) live cell imaging (Fig. 1) and importantly, CIZ1 AB cells reversed this effect and restored G1/S transition timing in post-quiescent cells, demonstrating CIZ1 regulates G1 length and timing for S-phase entry (Fig. 5C and Supplementary Fig. S8). Similarly, in post-quiescent cells, the proportion of cells entering S-phase after serum removal showed that 15 h after release CIZ1 KO cells efficiently initiate DNA replication, whereas WT and CIZ1 AB cells do not efficiently enter S-phase (Fig. 5D).

CIZ1 is a key regulator of cyclin A localization on chromatin and the nuclear matrix [14, 15]. The localization of cyclin A to chromatin is mediated via a cyclin binding box (Cy-box) CIZ1 [14] and regulated by post-translationally through CDK mediated phosphorylation of CIZ1 [15]. Mutation of the Cy-ii box or hyperphosphorylation of CIZ1 both prevent CIZ1 DNA replication function, with a concurrent loss of cyclin A chromatin/NM recruitment [14, 15]. Here the effect of CIZ KO on cyclin A localization was performed in post-quiescent cells (Fig. 5E and F). This revealed no difference in cyclin A or cyclin E expression levels between WT and CIZ1 KO cells 20 h post-release, although there appears to be lower cyclin expression in CIZ1 AB. However, CIZ1 KO cells were found to have a reduction in chromatin associated cyclin A, and this effect is effectively reversed after ectopic CIZ1 expression (Fig. 5G), showing that CIZ1 is required to localize cyclin A–CDK2 to chromatin. This reduction of cyclin A on chromatin is consistent with the observed role for CIZ1 promoting cyclin A–CDK2 localization at the G1/S transition that is required for efficient initiation DNA replication [14, 15].

### CIZ1 KO cells require higher CDK activity to initiate DNA replication

**Figure 6.**
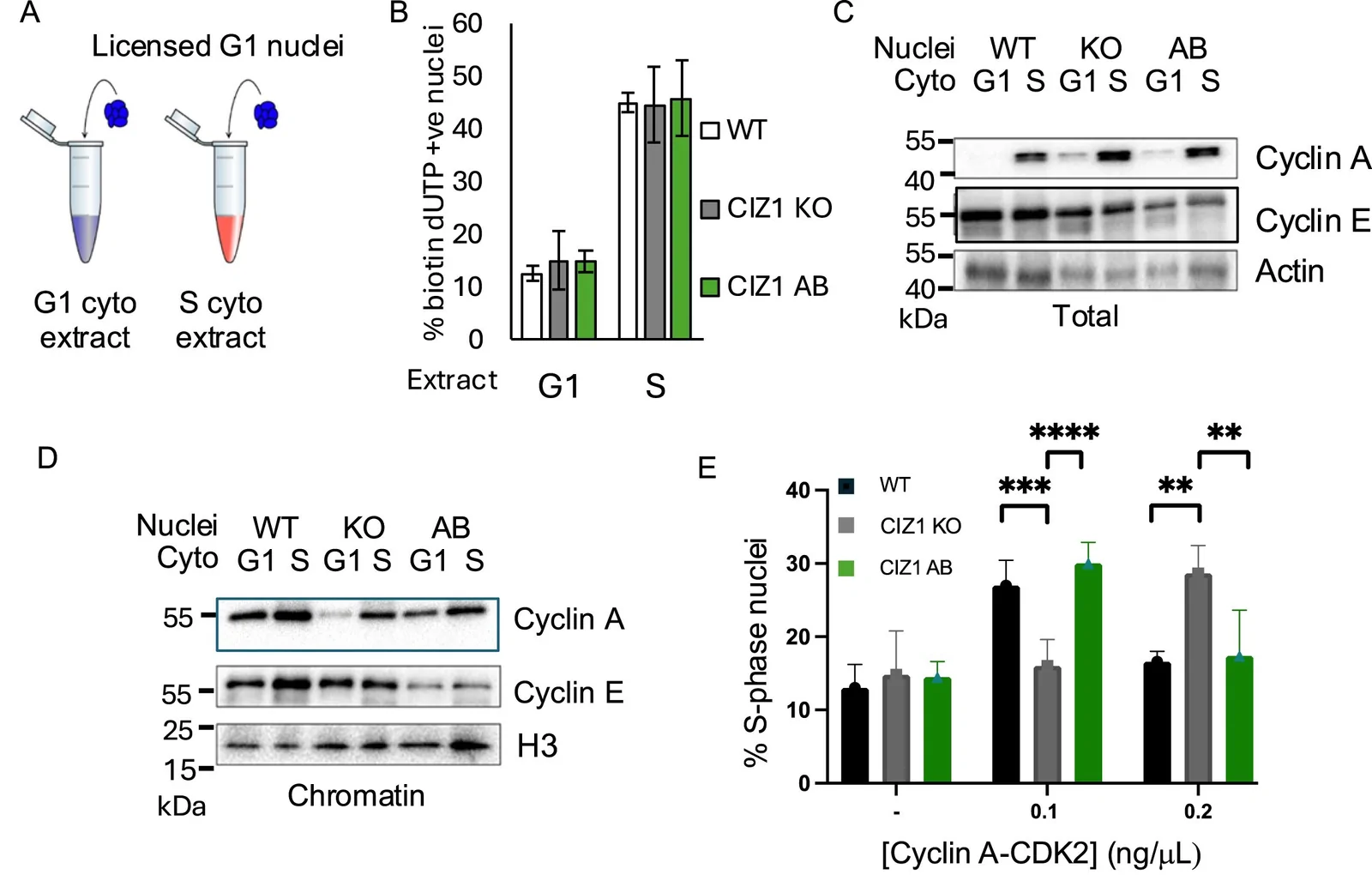
CIZ1 KO cells require increased cyclin A–CDK2 activity to promote initiation of DNA replication. (**A**) Schematic of *in vitro* cell free reactions using licensed late G1 nuclei and S-phase cytosolic extracts. (**B**) Initiation of DNA replication in WT, CIZ1 KO, and CIZ1 AB nuclei in S phase (Biotin-16-dUTP positive) after 30-min incubation in G1, and S phase extracts. Showing mean ± S.D where *n* = 3. (C, D) Western blots of total protein (**C**), and chromatin bound fractions (**D**) showing cyclin E and cyclin A (lower band indicated with arrow) after incubation in G1 and S phase extracts. (**E**) Proportion of WT, CIZ1 KO and CIZ1 AB nuclei in S phase after incubation with recombinant Cyclin A–CDK2 at 0, 0.1 and 0.2 ng/μl indicated concentrations. Showing mean ± S.D where *n* = 3. Statistical analysis was performed using one-way ANOVA with Tukey’s multiple comparisons test using GraphPad Prism software. Where ** = *P *< .01, *** =*P *< .001,. **** =*P *< .0001.

G1 length is a critical regulator of genomic stability. In post-quiescent cells, replication licensing is deficient predisposing cells to replication stress [49]. Similarly, cells with reduced G1 length are associated with partial licensing of DNA replication origins cells leading to DNA replication stress [44]. These data show both a reduction in G1 length in post-quiescent cells, suggesting that CIZ1 KO cells may have defects in replication licensing. Therefore, to further investigate the effects of CIZ1 KO on replication licensing, a cell free DNA replication assay was used. This approach utilizes DNA replication licensed nuclei to initiate DNA replication when stimulated by cyclin A–CDK2and allows for quantitative determination of the CDK activity to initiate DNA replication [14, 15, 16, 41, 50]. Initially, to investigate how CIZ1 KO affects DNA replication licensing, G1 nuclei were prepared from WT, CIZ1 KO and CIZ1 AB cells, and incubated in cytosolic S-phase extracts to promote initiation of DNA replication and S-phase nuclei determined by incorporation of biotin-dUTP (Fig. 6A, B). Nuclei that are in S-phase are identified in control reactions with G1 extracts and licensed nuclei, whereas initiation of DNA replication is shown by an increase in S-phase nuclei when incubated in S-phase cytosolic extracts. No observable differences in the proportion of nuclei that initiate DNA replication for WT, CIZ1 KO and CIZ1 AB cells, showing that there are no gross defects in replication licensing. However, reduced cyclin A protein levels and reduced chromatin loading was found in CIZ1 KO nuclei incubated in G1 cytosolic extracts relative to WT and CIZ1 AB nuclei (Fig. 6C and D). Cyclin A was present in the cytosolic fraction in CIZ1 KO cells, but this cyclin A pool did not efficiently load onto chromatin. Comparison of G1 and S-phase extracts in CIZ1 KO cells showed little cyclin A on chromatin in G1 extracts but in S-phase extracts cyclin A binds efficiently to chromatin to levels similar to WT and CIZ1 AB cells (Fig. 6D). CIZ1 KO nuclei with S-phase extracts showed promoted efficient chromatin loading as S-phase cytosolic extracts contain both CIZ1 and cyclin A–CDK2.

To determine how CIZ1 affects cyclin A localization *in vitro*, cell free DNA replication reactions were performed using G1 extracts supplemented with recombinant cyclin A and CIZ1, allowing precise control of their concentrations. There is a two-fold range within which cyclin A–CDK2 promotes initiation *in vitro* [14, 50]. To determine if WT, CIZ1 KO and CIZ1 AB nuclei licensed to replicate, nuclei were incubated with recombinant cyclin A–CDK2 *in vitro* at optimal (0.1 ng/μl). At this concentration, both WT and CIZ1 add back nuclei efficiently initiate DNA replication, but CIZ1 KO nuclei did not initiate DNA replication, showing that ectopic expression of CIZ1 (CIZ1 AB) effectively compensates for CIZ1 ablation. (Fig. 6E). As CIZ1 KO cells have higher intrinsic CDK activity, we reasoned that these nuclei may require increased CDK activity to initiate DNA replication. Using two-fold higher cyclin A–CDK2 levels (0.2 ng/μl) efficiently promotes initiation in CIZ1 KO cells, but this level this level cannot initiate DNA replication in WT and CIZ1 AB nuclei, identifying a shift in the CDK threshold and that WT and CIZ1 KO nuclei require distinct levels of CDK activity to promote initiation of DNA replication *in vitro*.

**Figure 7.**
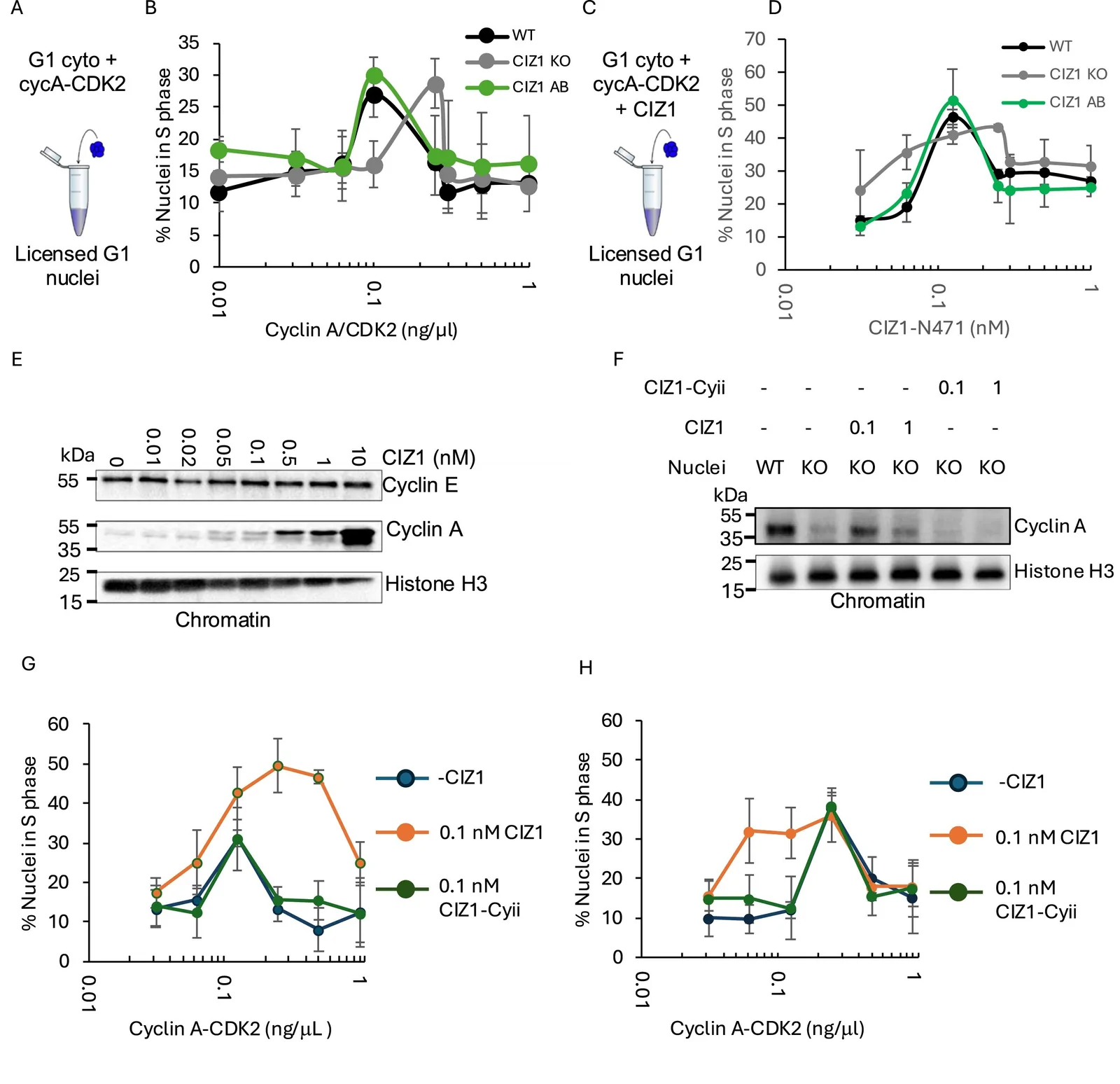
CIZ1 is required to localize cyclin A–CDK2 activity to chromatin to determine CDK threshold levels to initiate DNA replication. (**A**) Graphic experimental overview. (**B**) Cell free DNA replication assays showing the effect of increasing cyclin A–CDK2 in WT, CIZ1 KO and CIZ1 AB nuclei. (**C**) Experimental overview of (D) (**D**) Cell free DNA replication assays showing the effect of increasing CIZ1 concentrations in cell free reactions containing 0.1 ng/μl cyclin A–CDK2 in WT, CIZ1 KO and CIZ1 AB nuclei. (**E**) Immunoblot showing actin, cyclin E and cyclin A levels in chromatin fractions from cell free reactions using titration of recombinant CIZ1. CIZ1 promotes cyclin A binding to chromatin. (**F**) Immunoblot showing Histone H3 and cyclin A levels in chromatin fractions from cell free DNA replication assays with WT and CIZ1 KO nuclei as indicated. WT CIZ1 or CIZ1 Cy-ii (cyclin A binding deficient mutant) are added and effect on cyclin A chromatin loading is shown. (**G**) Effect of increasing CIZ1 levels in WT nuclei with recombinant CIZ1 or cyclin binding deficient mutant (CIZ1 Cy-ii), showing mean ± S.D where *n* = 3. (**H**) As for G using CIZ1 KO nuclei showing mean ± S.D where *n* = 3.

There are defined CDK thresholds that regulate the G1/S and G2/M transitions [36, 51–53]. Our data suggest that this CDK threshold may be dysregulated in CIZ1 KO cells and nuclei. To quantitatively determine the kinase thresholds that promote initiation, titration of recombinant cyclin A–CDK2 was performed in cell free DNA replication assays using WT, CIZ1 KO and CIZ1 AB nuclei (Fig. 7A). Cyclin A–CDK2 promotes initiation of DNA *in vitro* at precise CDK activities and shows distinct concentration dependent effects with initiation of DNA replication occurring within a narrow ∼2-fold range of cyclin A–CDK2 activity (Fig. 7B).

In WT nuclei, cyclin A–CDK2 promotes initiation of DNA replication at 0.1 ng/μl and two-fold higher levels prevent initiation of DNA replication (Fig. 7B). In contrast CIZ1 KO nuclei require two-fold higher concentrations of cyclin A–CDK2 to efficiently initiate DNA replication to WT levels (Fig. 7B). This level of CDK activity is non-permissive for DNA replication in WT nuclei and identifies that CIZ1 KO nuclei have an increased CDK activity threshold to initiate DNA replication. These results demonstrate that CIZ1 KO nuclei are replication licensed but require increased cyclin A–CDK2 activity to efficiently initiate DNA replication. These data suggest that CIZ1 modulates the CDK thresholds required for initiation of DNA replication and the G1/S transition.

Next to assess the levels of CIZ1 that are required to initiate DNA replication, CIZ1 was titrated into cell free DNA replication assays (Fig. 7C and D). This shows that WT and CIZ1 KO nuclei have similar concentration dependent effects, peaking at 0.1 nM CIZ1 N471. Whereas CIZ1 KO nuclei required higher levels of recombinant CIZ1 to efficiently initiate. Next to assess the effect of CIZ1 on cyclin A chromatin binding, CIZ1 was titrated into WT or CIZ1 KO nuclei. This revealed that CIZ1 efficiently increases cyclin A chromatin binding in a concentration dependent manner (Fig. 7E) suggesting that levels of chromatin bound cyclin A is modulated by CIZ1.

To investigate if cyclin binding is required to modulate the CDK threshold levels that promote initiation of DNA replication, WT CIZ1 or a cyclin binding deficient CIZ1 mutant (CIZ1 Cy-ii) [14] were titrated into cell-free DNA replication reactions and cyclin A localization to chromatin assessed (Fig. 7F). WT nuclei have high levels of cyclin A on chromatin, whereas CIZ1 KO nuclei have very low cyclin A chromatin binding. CIZ1 enhances cyclin A chromatin loading in CIZ1 KO nuclei and this effect is dependent on cyclin binding as this effect is absent with the cyclin binding deficient CIZ1 Cy-ii mutant (Fig. 7F). To identify if cyclin binding is a key regulator to promote initiation of DNA replication, WT nuclei were incubated with increasing cyclinA–CDK2 activity with or without CIZ1 or CIZ1 Cy-ii. CIZ1 increases the permissive range of cyclin A–CDK2 activity that promotes DNA replication, suggesting that it modulates CDK thresholds at the G1/S transition and this effect is dependent on cyclin binding as this effect is absent in the cyclin binding CIZ1 mutant (Fig. 7G). Titration of cyclin A–CDK2 in CIZ1 KO nuclei showed that a two-fold higher concentration of cyclin A–CDK2 (0.2 ng/ml) is required to initiate DNA replication. The requirement for a higher cyclin A–CDK2 level is reversed by addition of recombinant CIZ1, but not by the cyclin binding deficient mutant, demonstrating that binding and localization of cyclin A–CDK2 is required for optimal initiation of DNA replication (Fig. 7H). Importantly, the reduced levels of chromatin associated cyclin A can be increased by ectopic expression of CIZ1, or addition of recombinant CIZ1 protein showing that CIZ1 is necessary for efficient cyclin A chromatin loading. Significantly, the CIZ1 cyclin binding domain is required to optimize initiation of DNA replication, suggesting that CIZ1:cyclin A interactions and their stoichiometry are important regulators of the CDK threshold level that promotes the G1/S transition.

### CIZ1 requires cyclin A binding to prevent DNA replication stress

**Figure 8.**
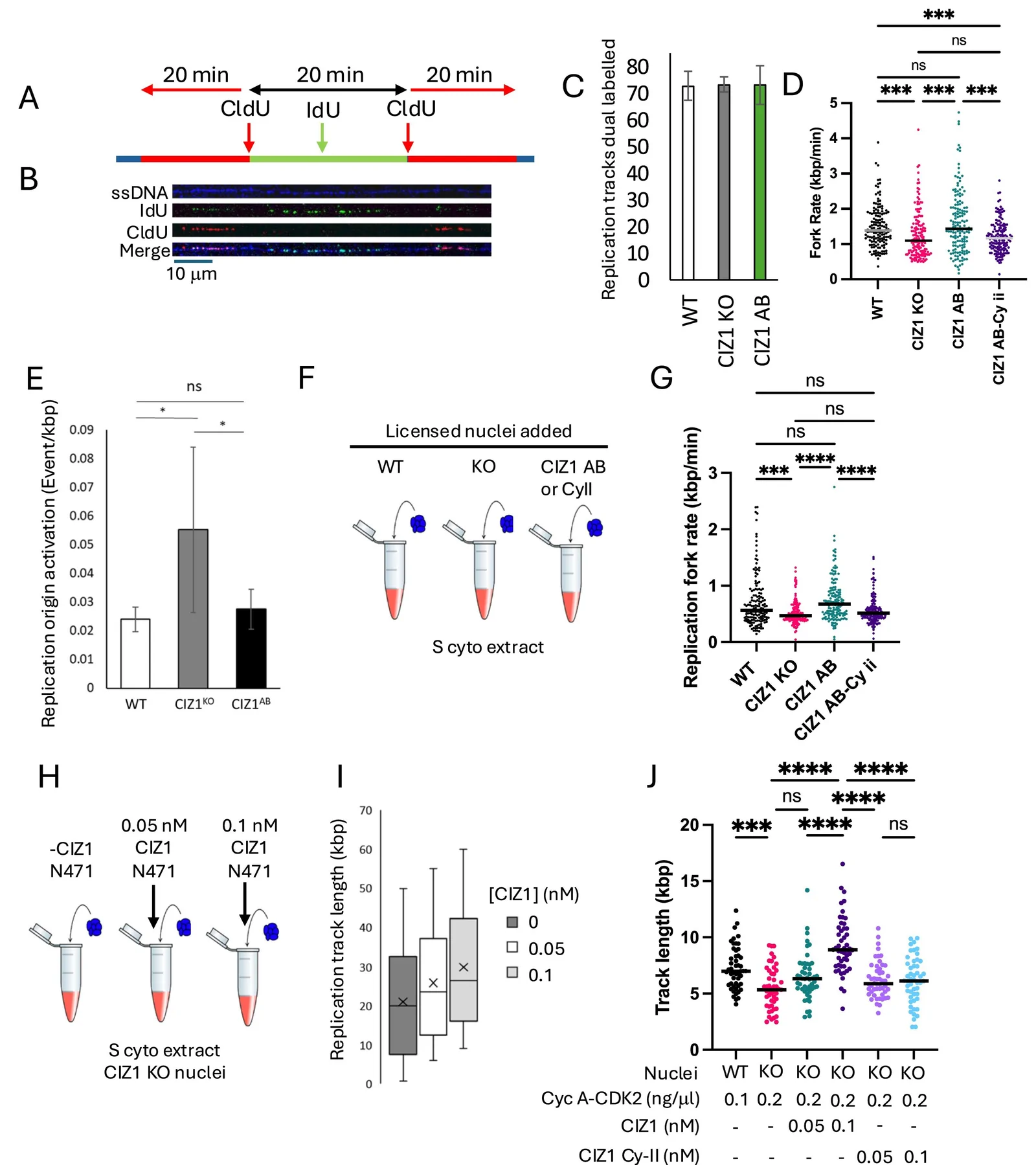
CIZ1 ablation promotes DNA replication stress. (**A**) Experimental overview of DNA labelling procedure for DNA combing. (**B**) Representative combed single-stranded DNA (ssDNA), CldU and IdU labelled DNA. (**C**) The percentage of WT, CIZ1 KO, and CIZ1 AB DNA fibres dual labelled after 2 consecutive 20-min incubations in IdU and CldU. (**D**) DNA replication fork rates were determined for WT, CIZ KO and CIZ1 AB cells. Data show individual fork rates, where *n* = 150. Nonparametric data were analysed by ANOVA using the Kruskal–Wallis’s test and Dunn’s multiple comparisons test, where *=*P *< .05, ** = *P *< .01, *** =*P *< .001 and **** = *P *< .0001. (**E**) DNA replication origin firing was determined per kbp of DNA, with standard deviation, where *n* = 150 for all cells. Nonparametric data were analysed by ANOVA using the Kruskal–Wallis’s test and Dunn’s multiple comparisons test. Where *=*P *< .05. (**F**) Cartoon of *in vitro* DNA replication assays using WT, CIZ1 KO and CIZ1 AB nuclei respectively in S-phase cytosolic extracts. (**G**) Data show individual fork rates for each condition, where *n* = 151 for WT, 151 for KO, 154 for CIZ1 AB and 151 for CIZ1 Cy-ii. Statistical analysis was performed using non-parametric ANOVA analysis using the Kruskal–Wallis’s test and Dunn’s multiple comparisons test. Where *=*P *< .05, ** = *P *< .01, *** =*P *< .001 and **** = *P *< .0001. (**H**) Cartoon of *in vitro* DNA replication assays using licensed G1 nuclei prepared from CIZ1 KO cells incubated in S-phase extracts supplemented increasing concentrations of CIZ1-N471 as indicated. (**I**) The length of replication tracks of WT and CIZ1 KO nuclei supplemented with increasing concentrations of CIZ1-N471 or CIZ1-N471-CyII-as indicated. Data shown in box whisker plots showing median, quartiles and standard deviation. J) DNA replication track length for WT and CIZ1 KO nuclei incubated for 30 min in G1 extracts with recombinant cyclin A–CDK2 and CIZ1 or CIZ1 Cy-ii mutation as indicated. Data show individual fork rates, where *n* = 50. Statistical analysis was performed using parametric two-way ANOVA analysis using the Tukey’s multiple comparisons test. Where *** =*P *< .001 and **** = *P *< .0001.s

CIZ1 ablation in murine models increases sensitivity to genotoxic stress [18], promotes cellular transformation [21] and tumorigenesis [8, 17]. Here, CIZ1 KO cells have been shown to have dysregulated CDK activity through overexpression of cyclin E, and require increased cyclin A-CDK activity to promote initiation of DNA replication. Dysregulation of CDK activity is associated with enhanced DNA replication stress [54, 55]. To assess if CIZ1 KO cells have increased DNA replication stress, DNA combing was used to determine replication fork rates and origin usage (Fig. 8A and B).

There were no differences in the frequency of dual labelled DNA replication events (Fig. 8C). However, the replication fork rate is significantly reduced in CIZ1 KO cell lines relative to the parental control cells (WT: 1.3 kbp/min, CIZ1 KO: 0.9 kbp/min, *P *< 0.001 Fig. 8C). This effect was reversed by ectopic expression of GFP-CIZ1 reversed (CIZ1 AB: 1.4 kbp/min, *P *< 0.001. Fig. 8D), demonstrating that CIZ1 regulates DNA replication fork rates. The reversal of DNA replication stress is dependent on cyclin binding, as the cyclin binding deficient CIZ1 mutant does not increase replication fork rates to WT levels, as seen for CIZ1 AB cells (Fig. 8D). Importantly, the loss of cyclin binding (CIZ1 Cy-ii) results in no significant differences between fork rates for CIZ1 KO nuclei with or without recombinant CIZ1 Cy-ii, consistent with CIZ1 modulating fork rates through cyclin binding and chromatin localization. To compensate for reduced fork rates, CIZ1 KO cells utilize cryptic origins increasing the frequency of replication origin firing to compensate for a reduction in fork speed in CIZ1 KO cells relative to WT and CIZ1 AB cells (Fig. 8E). These data demonstrate that CIZ1 KO cells display DNA replication stress that can be reversed by ectopic expression of CIZ1. Significantly, this reversal of the DNA replication stress phenotype requires cyclin binding to CIZ1 via its Cy-ii cyclin binding box.

To complement cell-based analysis, cell-free DNA replication assays were performed and replication dynamics analysed by DNA combing. This approach allows precise titration of CIZ1 or cyclin A–CDK2 and the effect on DNA replication determined. DNA combing assays were adapted for analysis for *in vitro* DNA replication assays (Fig. 8F). Critically, DNA combing from cell free experiments phenocopies cell-based experiments. CIZ1 KO nuclei produced shorter replication tracks than WT, or CIZ1 AB nuclei (Fig. 8G). Furthermore, in CIZ1 KO nuclei replication tracks could be lengthened by increasing CIZ1-N471 levels in cell free assays (Fig. 8 H,I) indicating that addition of recombinant CIZ1 protein could alleviate the DNA replication stress phenotype. To determine if this effect was mediated by recruitment of cyclin A–CDK2, CIZ1 Cy-ii mutant was titrated into reactions. The cyclin binding deficient mutant is unable to enhance fork rates demonstrating that cyclin binding is required to increase DNA replication fork rates and reduce DNA replication stress (Fig. 8J). These data collectively demonstrate that CIZ1 KO cells have increased DNA replication stress and that this effect could be reversed through titration of recombinant CIZ1 protein or through ectopic CIZ1 expression. In both contexts, cyclin recruitment is required to alleviate DNA replication stress, demonstrating that CIZ1 reduces DNA replication stress through binding and localization of cyclin A–CDK2. These data suggest that CIZ1 through interactions with cyclin A–CDK2 regulates DNA replication fork speeds and contributes to mechanisms that prevent DNA replication stress.

## Discussion

The cell cycle progression is driven by an increasing gradient of CDK activity [51]. The increasing gradient of CDK activity facilitates separation of replication licensing and initiation of DNA replication, and CDK activity peaks to promote mitosis [56, 57]. Intracellular CDK activity is regulated at numerous levels through transcriptional regulation of cyclin expression [28], phosphatase activity [58], regulatory kinase activity (CAK/WEE1), levels of CDK inhibitor proteins [29] and ubiquitin-proteasome activity [53]. These activities ensure that CDK activity is tightly regulated and coordinated within each phase of the cell cycle. Intracellular CDK activity reporters have identified that there are intrinsic CDK activity levels dictate exit from the cell cycle or the rate of G1 exit due to stochastic CDK activities or through molecular memory by high CDK inhibitor levels in response to genetic stress in previous cell cycle. This work has identified that CIZ1 contributes to mechanisms that control the G1/S transition and modulate the specific CDK activity required for efficient initiation of DNA replication.

Analysis of CIZ1 KO fibroblasts revealed that CIZ1 modulates G1 length and affects CDK activity thresholds for the initiation of DNA replication. CIZ1 KO fibroblasts have reduced G1 length (Fig. 1) and bypass restriction point significantly earlier than WT cells (Figs 2 and 3). This is mediated by increased cyclin E and cyclin A expression at earlier stages of G1 phase (Fig. 4 and Supplementary Fig. S4). This study has identified an increase in cyclin expression and intracellular CDK activity in post-quiescent cells. The mechanism that underlies these changes has not been identified. However, CIZ1 ablated mice have altered epigenetic programmes [21, 59] that could provide a mechanism that leads to an increase in cyclin expression in CIZ1 KO cells.

CIZ1 KO cells display reduced cyclin A chromatin binding (Figs 5 and 6) and increased requirement for cyclin A–CDK2 activity to promote initiation of DNA replication (Fig. 7). Dysregulation of CDK activity is known to promote DNA replication stress and this was also observed in CIZ1 KO cells, demonstrating that CIZ1 contributes to mechanisms that prevent DNA replication stress (Fig. 8). These observations provide insight into CIZ1 function and its role in prevention of DNA replication stress. These data also provide insight into the association between CIZ1 ablation and enhanced DNA replication stress, which may underpin CIZ1 tumour suppressor function [17].

Replication licensing begins as cells exit mitosis and APC/C-CDH1 is activated reducing intracellular CDK activity. The length of G1 phase is an important regulatory step, as reductions in length can prevent efficient licensing of the genome [49]. Here, G1 length was similar for cycling cells in cycling fibroblasts. However, in post-quiescent cells there is a marked difference in G1 length (15.9 and 12.1 h, respectively) that may affect DNA replication licensing. [49], however, no defects were found in replication licensing *in vitro*. In CIZ1 KO nuclei, G1 phase length was found to be reduced and paradoxically, that higher levels of CDK activity is required to promote initiation of DNA replication (Figs 6 and 7).

CIZ1 KO cells increase cyclin E and cyclin A expression results in increased RB phosphorylation consistent with increased CDK activity in post-quiescent cells CIZ1 KO cells. The increased replication stress in CIZ1 KO cells is associated with the earlier overexpression of cyclin E and cyclin A, leading to a truncated G1 phase. It is well established that Cyclin E overexpression has multifaceted effects including reduced G1 length, promoting under licensing of replication origins, nucleotide pool exhaustion, promoting DNA replication stress, sensitivity to checkpoint inhibitors and whole genome duplication [46, 55, 60, 61]. Here increased cyclin E and cyclin A expression was associated with DNA replication stress, with reduced fork rates and a differential CDK threshold for initiation of DNA replication.

The differential cyclin A–CDK2 activity required for initiation of DNA replication correlates with CIZ1 KO cells have reduced chromatin associated cyclin A. The reduction in cyclin A loading to chromatin can be reversed by ectopic CIZ1 expression *in vivo* or addition of recombinant CIZ1 *in vitro* and requires the cyclin binding domain of CIZ1. The observed fork rate can be enhanced *in vivo* and *in vitro* through expression or addition of recombinant CIZ1, and significantly, titration of CIZ1 levels *in vitro* led to a concentration dependent increase in fork rate. In sum, the data demonstrate that the interaction of CIZ1:cyclin A–CDK2 in G1 phase and at the G1/S transition is protective from DNA replication stress. We propose that CIZ1 modulates the CDK threshold that promotes efficient initiation of DNA replication and contributes to mechanisms that prevent DNA replication stress.

The quantitative model for CDK-mediated cell cycle regulation states that increasing CDK gradients are sufficient to ensure temporal control of the cell cycle [51, 52]. The best available evidence utilizes fluorescent CDK reporters to determine intracellular CDK activities through the cell cycle [27, 29]. The data presented here demonstrates that CIZ1 KO cells have enhanced CDK activity in post-quiescent cells (Fig. 3). These data demonstrated differential CDK activity in CIZ1 KO cells and WT cells, however, they do not provide a quantitative measurement of cyclin-CDK activity or levels. The precise levels of cyclin A–CDK2 required to promote initiation of DNA replication were quantitatively determined using an *in vitro* DNA replication assay that uses recombinant cyclin A–CDK2 to promote initiation of DNA replication in intact nuclei. These data show a paradoxical requirement for increased cyclin A–CDK2 levels in CIZ1 KO cells, despite the observation that they express G1/S cyclins at higher levels and reduce G1 length.

**Figure 9.**
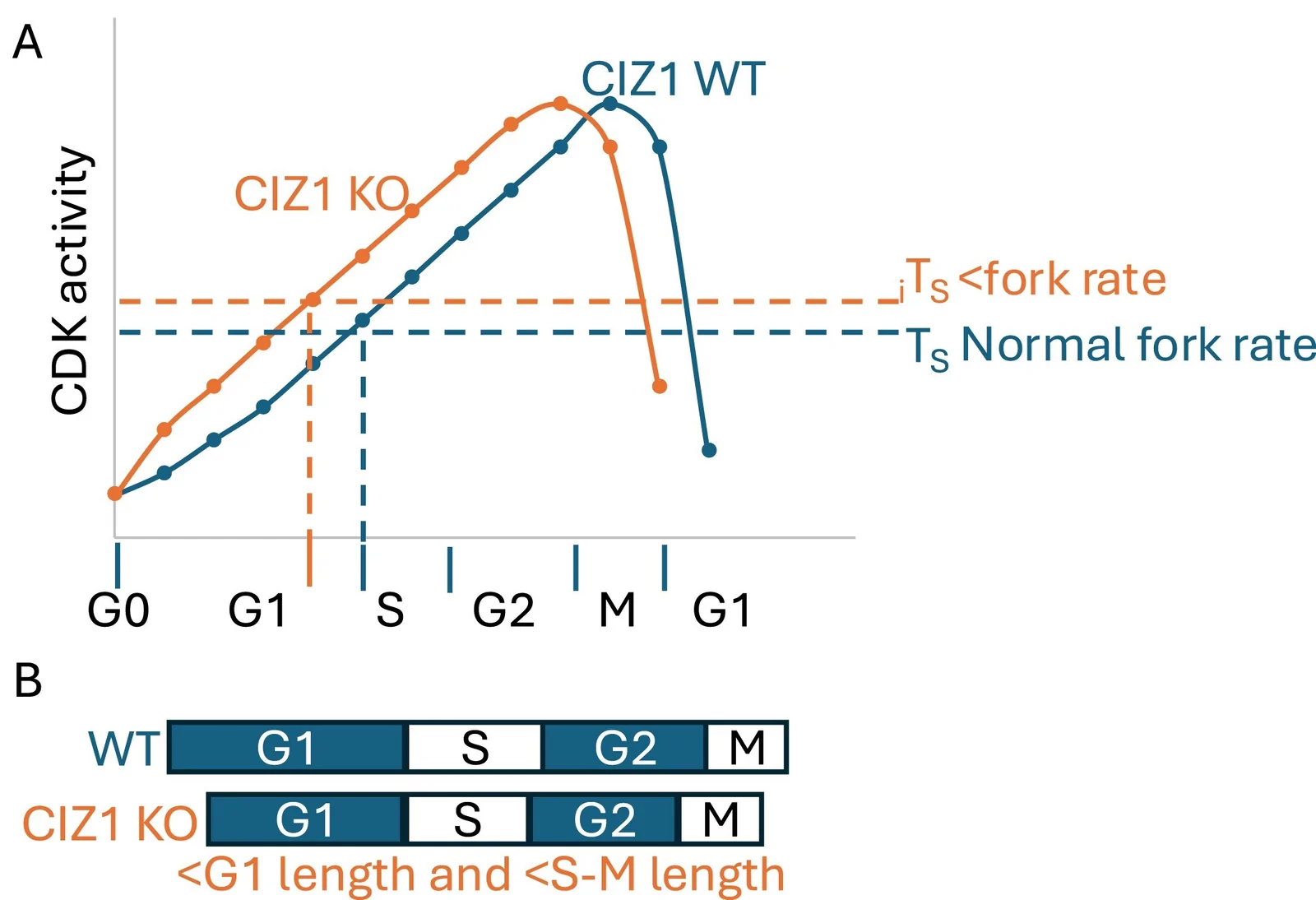
Model. CIZ1 modulates CDK thresholds to prevent DNA replication stress. (**A**). The quantitative model for CDK regulation of the cell cycle suggests that specific CDK thresholds regulate cell cycle transitions. The threshold for S phase entry and initiation of DNA replication is denoted T_S_ for WT cells. In CIZ1 KO cells, increased cyclin E and cyclin A expression results in increased CDK activity and a higher CDK activity threshold for initiation of DNA replication (_i_T_s_). In the absence of CIZ1, cells require a higher CDK activity to reach the threshold exit G1 phase. This is achieved through increasing CDK activity, resulting in reduced G1 length. This dysregulation of the CDK activity and mislocalization of cyclin A in G1 phase promotes DNA replication stress in CIZ1 KO mutants. (**B**) Differential cell cycle length in CIZ1 KO and WT cells. The increase in CDK activity in CIZ1 cells reduces G1 length and overall cell cycle length relative to WT cells, primarily through reduction in G1 length.

CIZ1 KO cells are deficient in cyclin A–CDK2 chromatin localization (Figs 5F and 6C). We propose that cells compensate for CIZ1 loss by over-expressing cyclins E and A, that may be mediated through changes in epigenetic regulation in CIZ1 KO cells [19, 21]. In addition, CIZ1 KO cells utilize a higher CDK activity for transition of the G1/S transition as evidenced by higher intrinsic CDK activity (Fig. 2) and by the biochemical necessity of two-fold higher levels of cyclin A–CDK2 to initiate DNA replication *in vitro* (Fig. 7). Furthermore, addition of purified CIZ1 restores the optimal CDK threshold for initiation of DNA replication (Fig. 7) and DNA replication fork function (Fig. 8). Therefore, we have shown by multiple complementary approaches that CIZ1 regulates the CDK threshold at the G1/S transition. These data led to the following model, where CIZ1 is a key factor that determines the threshold for initiation of DNA replication and the transition from G1 to S phase (Fig. 9). In the absence of CIZ1, G1 length is reduced in post-quiescent cells and interestingly, the reduction in G1 length is associated with an increased threshold for initiation of DNA replication relative to WT cells. This dysregulation of CDK activity promotes DNA replication stress in CIZ1 KO cells and potentially provides a mechanism for the reduced genome stability as seen in murine models [17, 18, 19, 21].

CIZ1 has roles in both cell cycle regulation and epigenetic maintenance. This study has identified an increase in cyclin expression and intracellular CDK activity in post-quiescent cells. The mechanism that underlies these changes has not been identified. However, there is a potential regulatory mechanism through alterations in the epigenetic programme in the absence of CIZ1 [21, 59]. CIZ1 ablation results in alterations to the epigenetic landscape and increases propensity to generate genetic damage [9, 13, 19, 21, 62–64]. At present there are 2 distinct roles for CIZ1 in epigenetic regulation. CIZ1 interacts with the lncRNA XIST that contributes to mechanisms that mediate X-chromosome inactivation and the localization of the inactive X-chromosome (Xi) [8, 9, 19, 62, 63]. In addition, it has been noted that CIZ1 also contributes to the level of polycomb repressor complex 1 and 2 (PRC1/2) mediated H2AK119Ub and H3K27me3 [19]. CIZ1 KO cells show defects in chromosome condensation on entry into quiescence due to reduced histone 4 mono-methylation (H4K20me1). This led to an increase in expression of homology directed repair genes and an increase in activated Chk1 kinase and gammaH2AX levels in CIZ1 null mice [21].

The results here complement and extend previous observations and enhance mechanistic understanding of the observed genome instability in CIZ1 ablated mice. Intrinsic DNA replication stress observed in CIZ1 KO cells, and the reduction in replication fork rate seen *in vivo* and *in vitro* suggest that CIZ1 plays an important role in replication fork progression via prevention or alleviation of DNA replication stress. The increased fork rate observed in CIZ1 KO cells when CIZ1 is ectopically expressed could indicate that global epigenetic remodelling has occurred, alleviating DNA replication stress. However, *in vitro* DNA replication assays found that addition of CIZ1 protein increased DNA replication fork rates in a concentration dependent manner, suggesting that CIZ1 is exerting its effect acutely. Second, as fork rates are not affected by the cyclin binding deficient CIZ1 Cy-ii mutant, this suggests that CIZ1 affects fork rate specifically through recruitment and localization of cyclin A–CDK2. These data demonstrate that CIZ1 can only alleviate DNA replication stress through cyclin A–CDK2 binding and recruitment of cyclin A to chromatin to alleviate DNA replication stress. These data do not exclude a role for CIZ1 in modulation of the epigenetic landscape and regulation of DNA replication fork progression in CIZ1 null cells. However, the data identify that CIZ1 mediated cyclin A–CDK2 localization is a key regulator of DNA replication fork progression, thereby preventing DNA replication stress. The role of CIZ1 at either the replication fork or during the initiation phase is still to be determined. Taken together, these data suggest that CIZ1 maintains genomic stability through regulation of the CDK threshold that promotes initiation of DNA replication and prevention of DNA replication stress.

## Supplementary Material

gkag878_Supplemental_Files

## Data Availability

All data are available at FigShare: https://doi.org/10.6084/m9.figshare.31037887.
