# Supplementary material for: CIZ1 regulates G1 length and the CDK threshold for initiation of DNA replication to prevent DNA replication stress
Source: Nucleic Acids Res. 2026 Sep 12;54(17):gkag878. doi: 10.1093/nar/gkag878 (PMC13569497; doi:10.1093/nar/gkag878)
Supplement: Supplementary file 1 [file gkag878_supplemental_files.zip › NAR supplementary figures revised final.pdf]

Supplementary Figure 1

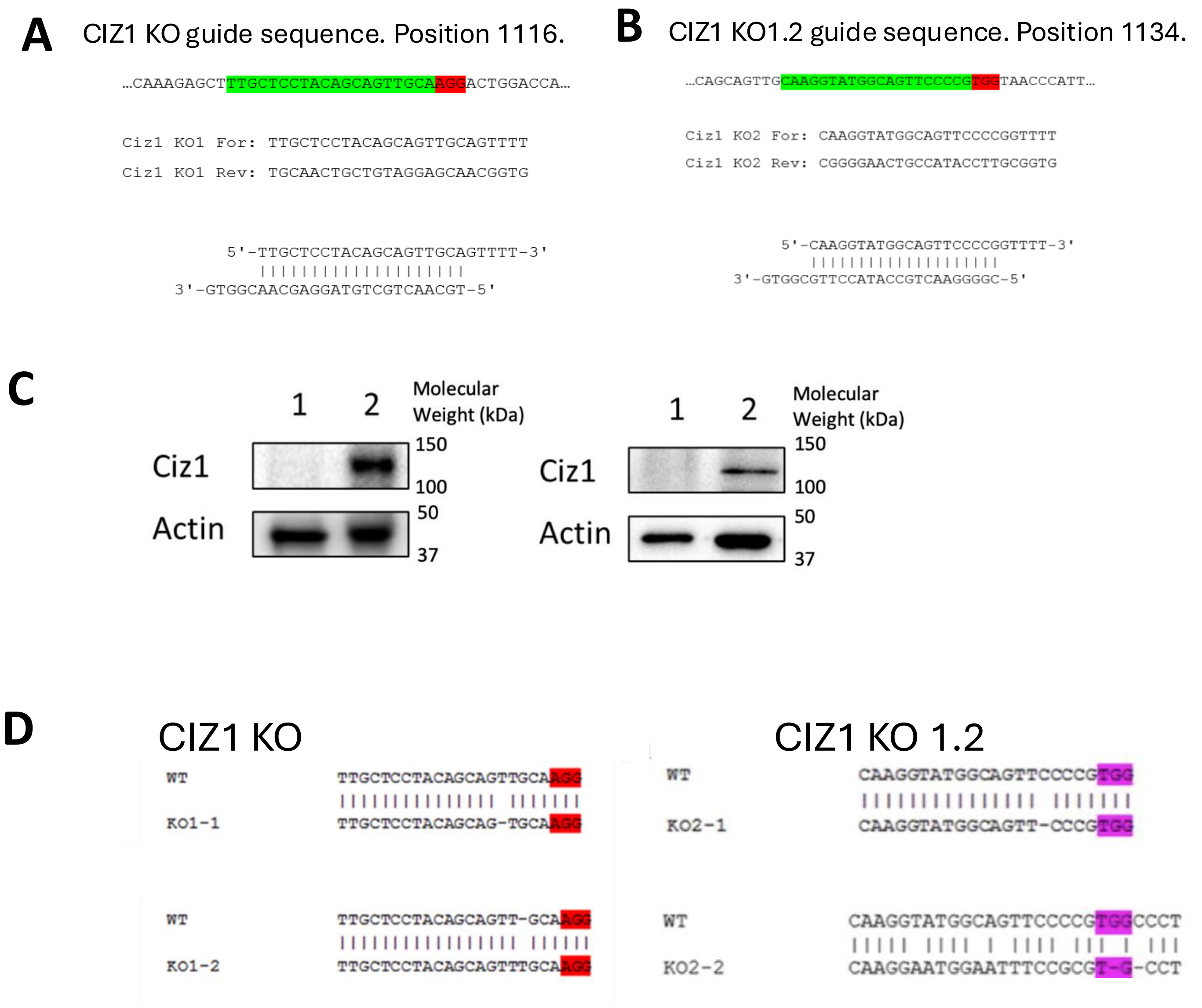

Figure S1. CRISPR-Cas9 mediated CIZ1 KO and CIZ1 KO1.2. A) Guide RNA sequence for CIZ1 KO. B) Guide RNA sequence for CIZ1 KO1.2 C) Western blot of CIZ1 levels in WT and CIZ1 KO cell lines. D) Nested PCR amplification of CIZ1 gene around the CRISPR-Cas 9 mediated cleavage site. Frame shift mutations are shown. PAM sequences are highlighted for CIZ1 KO and CIZ1 KO1.2

Supplemental Figure 2

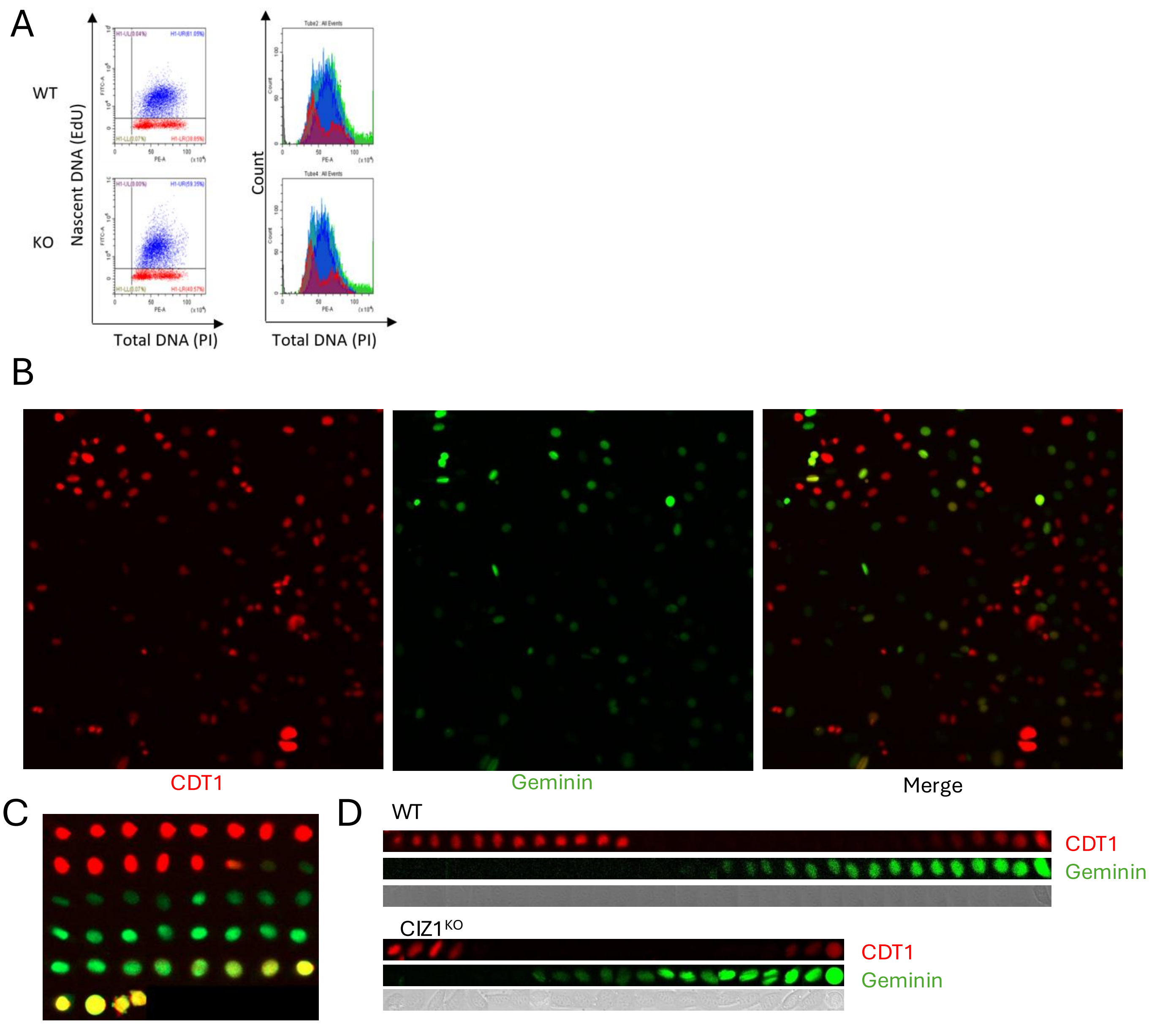

Figure S2. Live cell imaging of FuCCi labelled WT and CIZ1 KO cells. A) Flow cytometry of asynchronous murine fibroblasts labelled with EdU and PI. B) Representative Fucci labelled asynchronous fibroblasts, G1 reporter CDT1 (red) S/G2 reporter geminin (green) shown. C) Timelapse image of a single cell going through a complete cell cycle. D) Comparison of WT and CIZ1 KO cells post quiescence followed for a complete cell cycle. CIZ1 KO cells have a reduced cell cycle length relative to WT cells.

Supplemental Figure 3

A

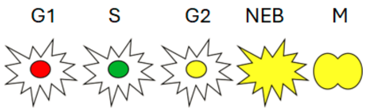

B

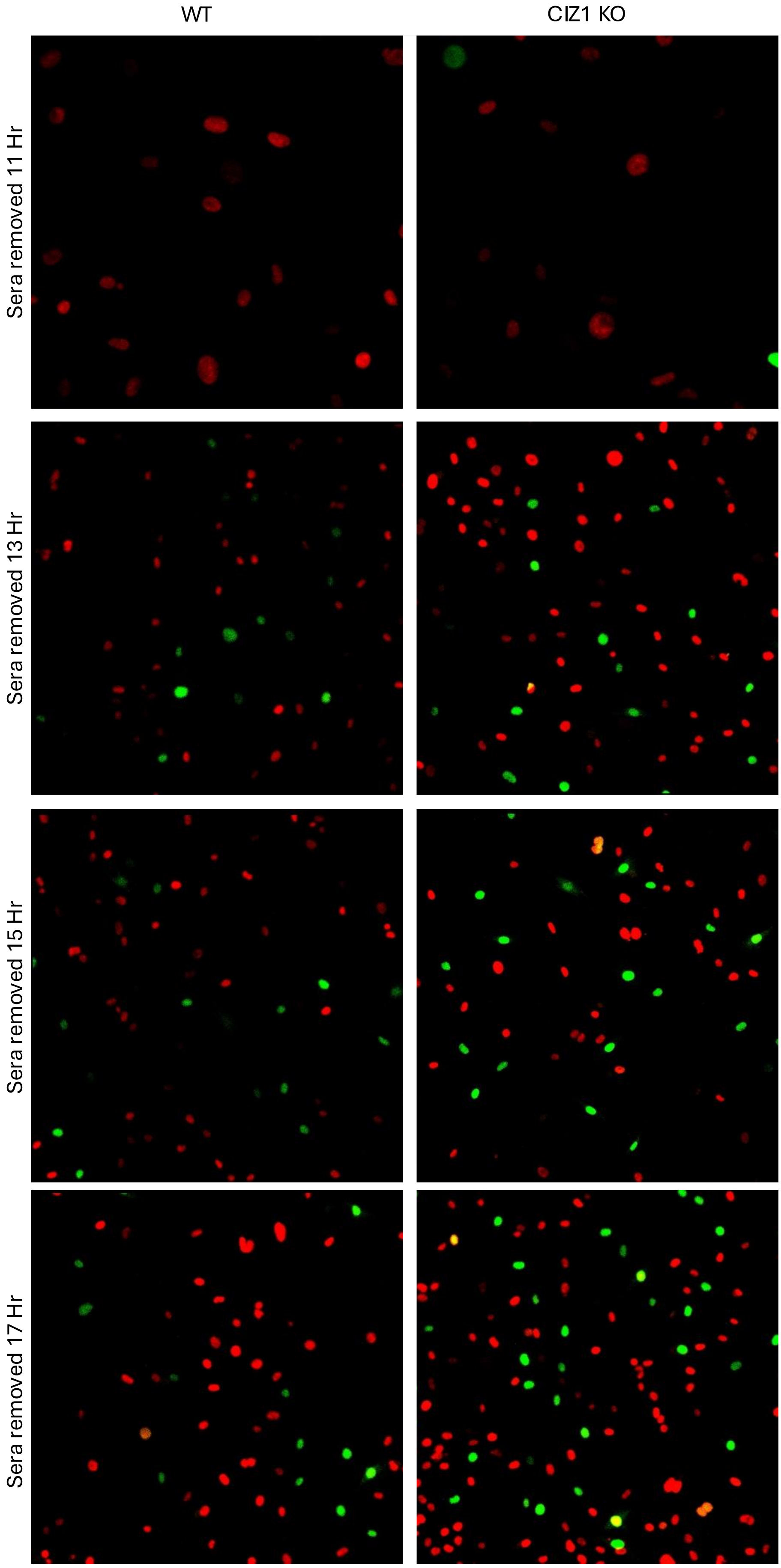

Supplemental Figure 3

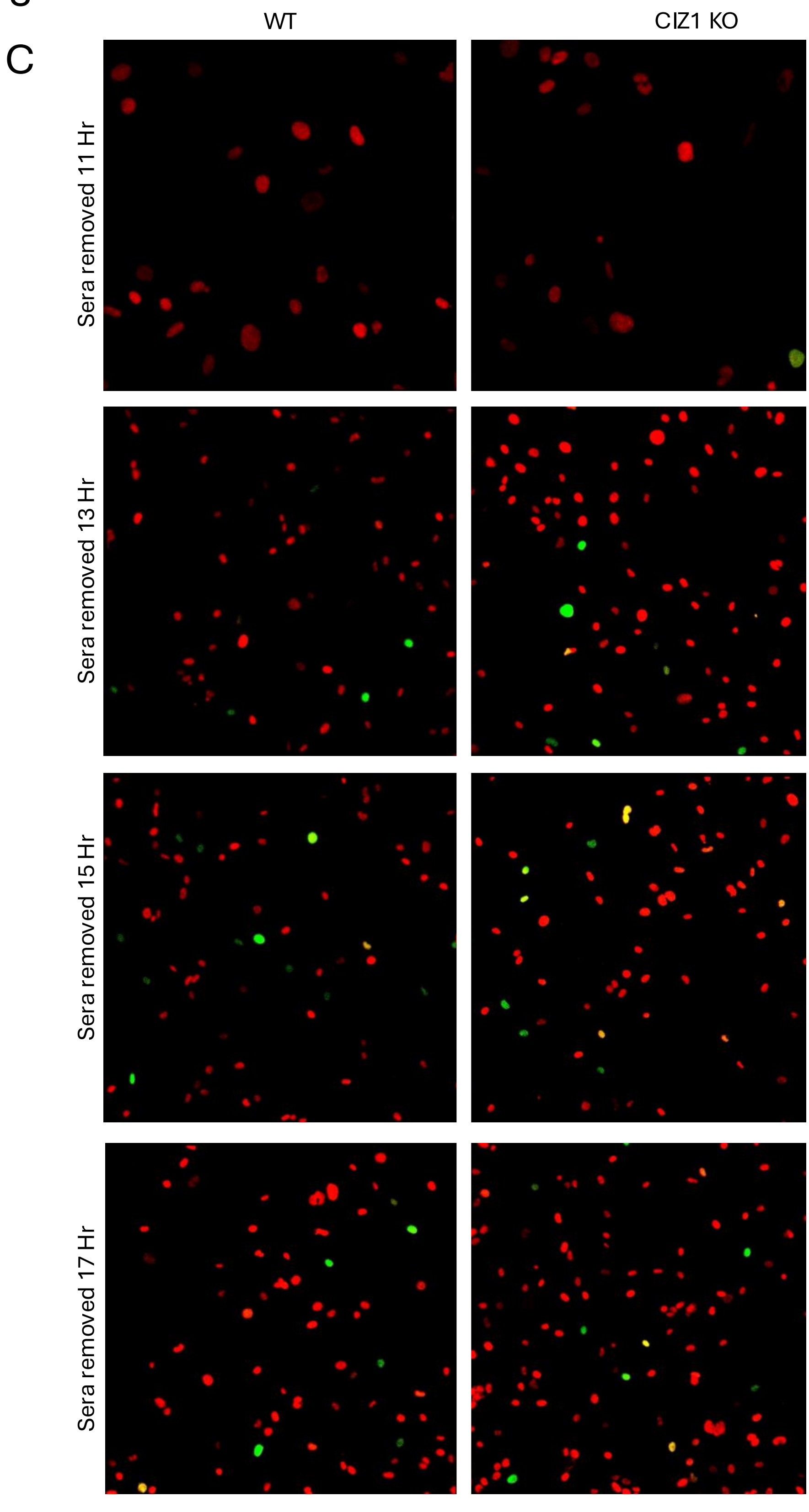

Figure S3. Representative images of post-quiescence cells after serum removal to identify restriction point. A) Cartoon of Fucci(CA) labelled cells with cell cycle stage. B) Representative still images of FuCCI labelled WT and CIZ1 KO cells with serum removed at indicated time points (11, 13, 15, 17 hours post release from quiescence). Images were taken 27 hours after release from quiescence. C) Embedded merged video of WT and CIZ1 KO cells labelled with FuCCi(CA) where serum has been removed at indicated time points.

Supplemental Figure 4

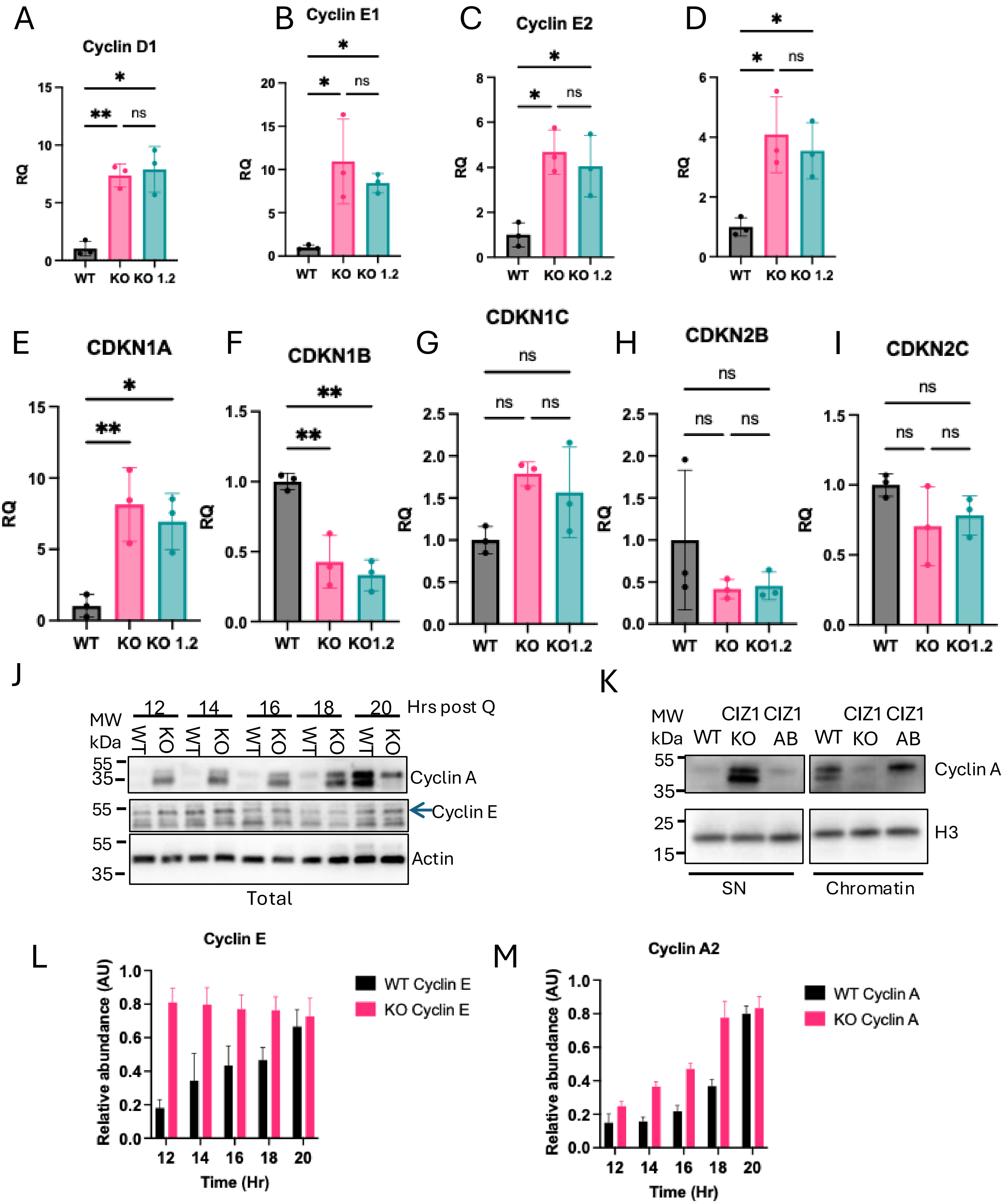

Figure S4. Cyclin Expression is increased in CIZ1 KO cells. A) Quantitation of mRNA levels in asynchronous, cycling WT, CIZ1 KO and CIZ1 KO1.2 for *CCND1* (A), *CCNE1* (B), *CCNE2* (C), *CCNA2* (D), *CDKN1A* (E), *CDKN1B* (F), *CDKN1C* (G), *CDKN2B* (H) and *CDKN2C* (I). J) Cells were harvested at time points after release from quiescence for quantitation of total protein levels for WT and CIZ1 KO cells showing western blot of cyclin A, Cyclin E and actin. K) CIZ1 promotes cyclin A chromatin binding. Detergent soluble protein and chromatin bound proteins are shown for cyclin A and Histone 3. L) Quantitation of data from Figure 4 F showing cyclin E levels in WT and CIZ1 KO cells in post-quiescent cells with time of protein harvest after release from quiescence. Data show average of 3 biological repeats with standard deviation. M) As for L, showing quantitation of cyclin A. Data show average of 3 biological repeats with standard deviation.

Supplementary Figure 5

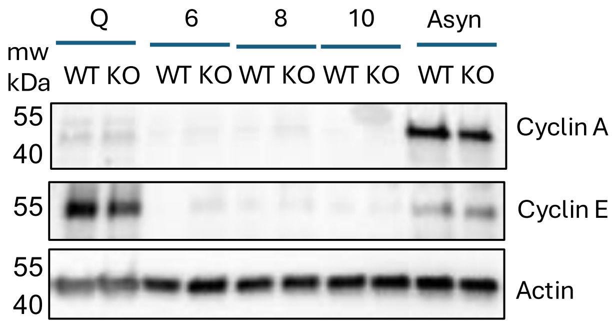

Figure S5. Relative expression of cyclin E and cyclin A in quiescent cells. Western blot of quiescent and released cells at indicated time points showing cyclin E, cyclin A and actin protein levels. For comparison, asynchronous (Asyn) cells are also shown.

Supplemental Figure 6

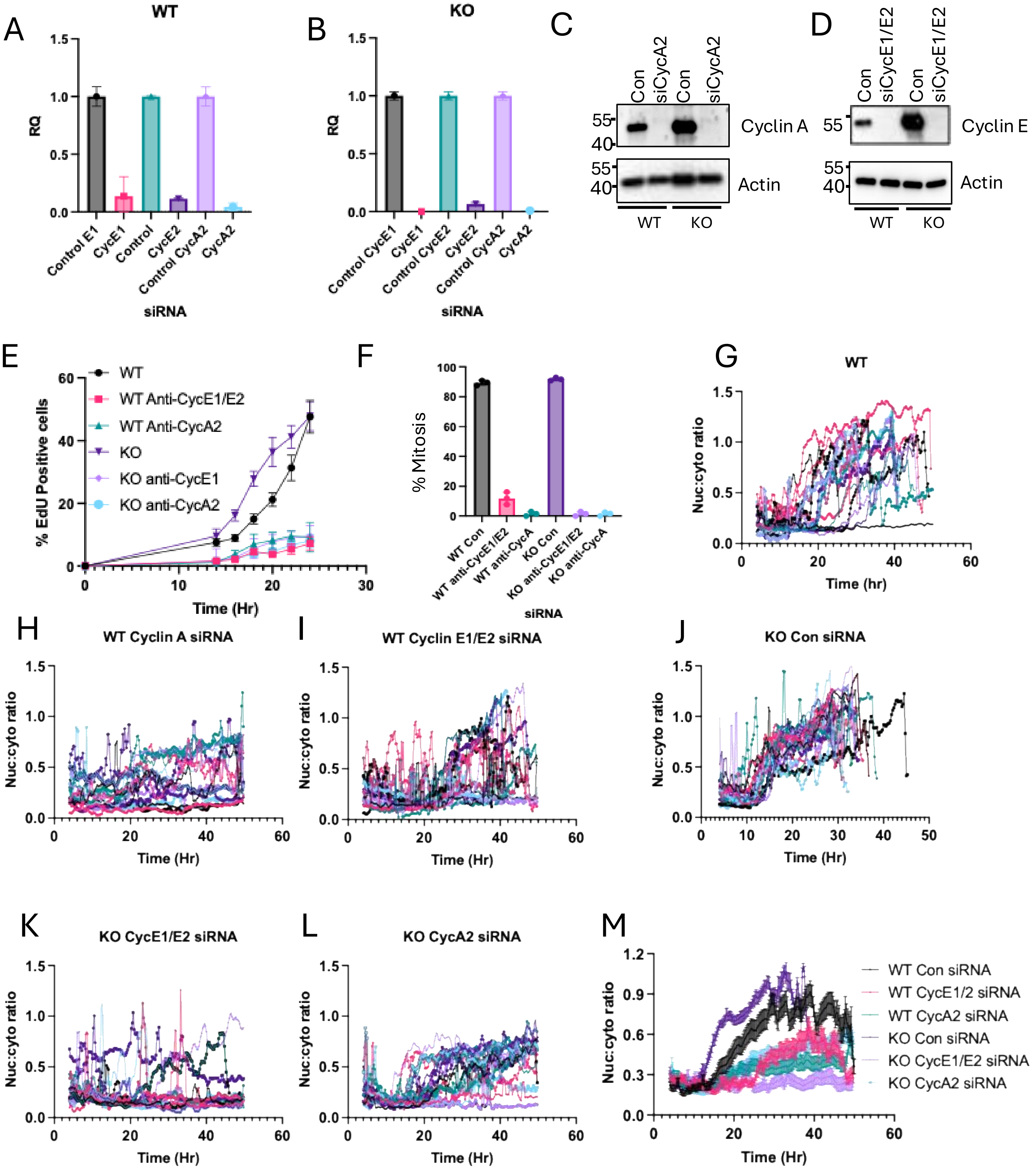

- **Figure S6. Depletion of CCNE1/CCNE2 or CCNA2 blocks cell cycle re-entry in post-quiescent cells.** A, B) qRT-PCR showing reduction in CCNE1/E2 or CCNA2 after siRNA mediated depletion in WT (A) and CIZ1 KO cells (B), C as for A showing cyclin E protein levels by immunoblot. D) as for B showing cyclin A2 protein levels by immunoblot. E) Percentage of cells in S-phase as shown by EdU labelling. Depletion of both cyclin E1/E2 or Cyclin A2 prevent S-phase entry. F) Percentage of cells that reach mitosis within 50 hours of quiescence release and siRNA treatment in WT and CIZ1 KO cells. G) Ratiometric analysis of intracellular CDK activity for WT cells released from Quiescence and treated with control siRNA. H) As for G treating cells with CCNE1/CCNE2 siRNAs, I) As for G, treating cells with CCNA2 targeting siRNA. J) CIZ1 KO cells treated with control siRNA and release from quiescence showing ratiometric nucleus to cytoplasmic fluorescence signal. K) As for J, but cells treated with CCNE1/CCNE2 targeting siRNAs. L) As for J, but with CCNA2 targeting siRNA. M) Averaged ratiometric CDK activity for WT and CIZ1 KO cells treated with control, CCNE1/CCNE2 or CCNA2 siRNAs. Data show an average of 20 cells from 3 biological replicates.

Supplementary Figure 7

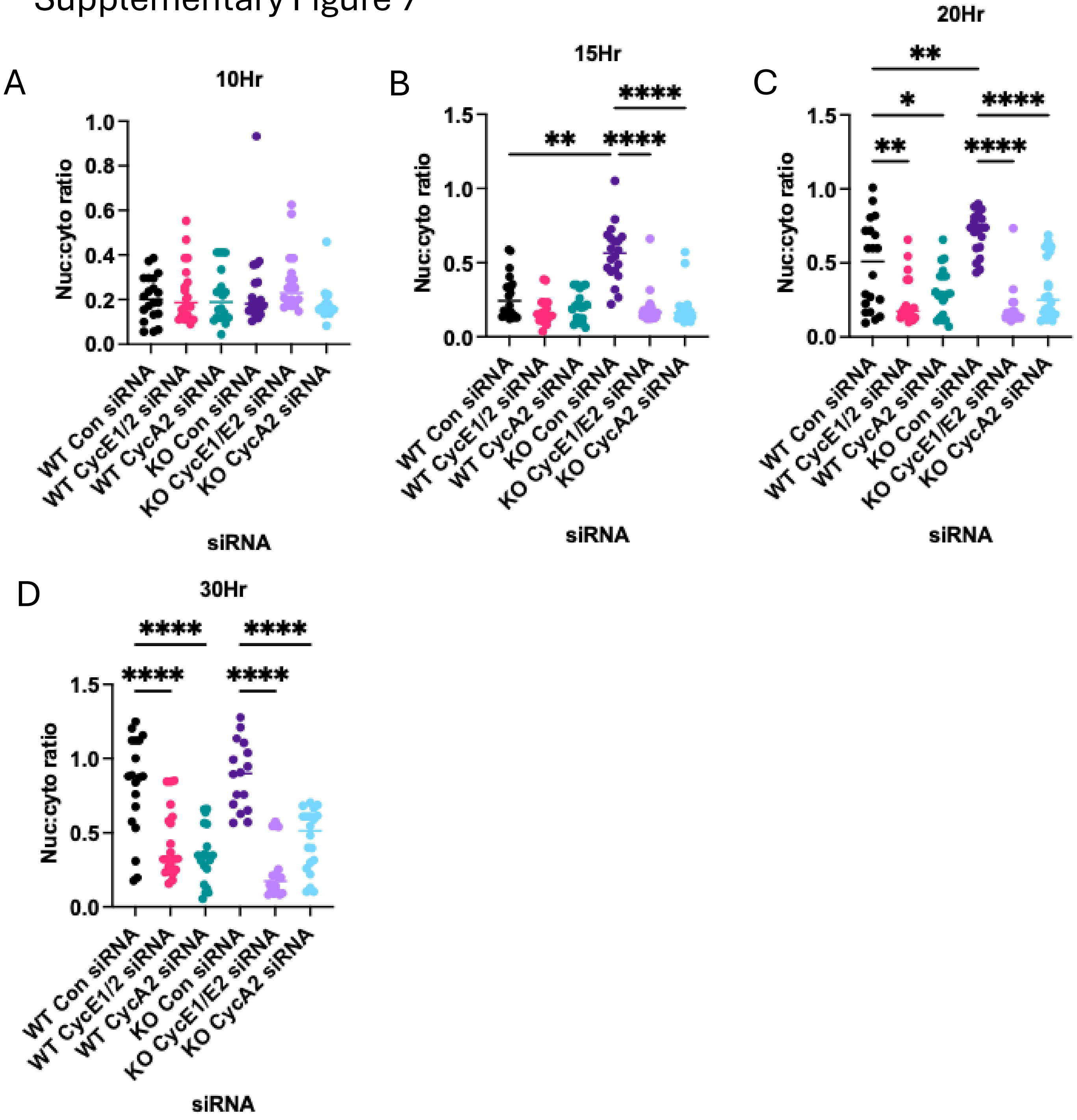

Figure S7. Analysis of the intracellular CDK activity during cell cycle re-entry. A-D. The ratio of cytoplasmic to nuclear fluorescence in WT and KO cells treated with control, *CCNE1/CCNE2* or *CCNA2* siRNAs. The ratio of cytoplasmic and nuclear fluorescence is shown at 10 (A), 15 (B), 20 (C) and 30 (D) hours post-release from quiescence and transfection with siRNA. Data shown were parametric for A,B and D with statistical testing performed using One-way ANOVA with Tukey's multiple comparisons test where *=$p<0.05$, ** = $p<0.01$, and **** = $p<0.0001$. C was non-parametric and analysed using One-way ANOVA using Krustal-Wallis multiple comparison test, where **** = $p<0.0001$.

Supplemental Figure 8

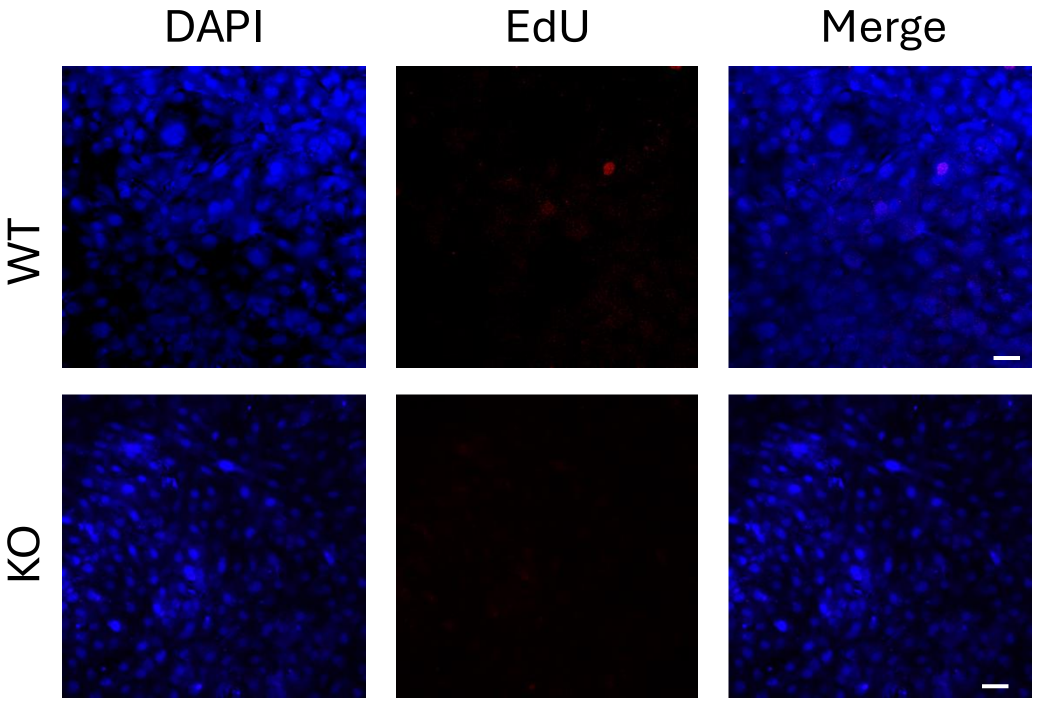

Figure S8. EdU labelling of confluent cells. DNA (blue) nascent replication (red) and merged images. EdU positive cells shown in red, with <1 % positive. White arrow highlights positive nuclei. The white bar represents the scale bar = 50μm.

Supplementary video files.

Supplementary video 1. Intracellular CDK sensor data for WT cells. Merged file of WT cells post-release from quiescence. Each frame is taken 15 minutes apart, composite of mNeonGreen-DHB and H2B-mScarlet. Also show single channel video for DHB and H2B-mScarlet.
Supplementary video 2, as for Supplementary video 1, but showing CIZ1 KK cell line.
Supplementary video 3, as for supplementary video 1, but showing CIZ1 1.2 KO cell line.

Supplementary Video 4-9. Intracellular CDK sensor data for WT and CIZ1 KO cells lines after siRNA mediated depletion of Cyclin E or Cyclin A or with control siRNA.

Video 4 WT cells with control siRNA showing single channels and merge.
Video 5 WT cells transfected with anti-CycA2 siRNA single channels and merged.
Video 6 WT cells transfected with anti-CycE1/E2 siRNA single channels and merged.
Video 7 CIZ1 KO cells with control siRNA showing single channels and merge.
Video 8 CIZ1 KO cells transfected with anti-CycE1/E2 siRNA single channels and merged.
Video 9. CIZ1 KO cells transfected with anti-CycA2 siRNA single channels and merged.

Full Western blots

Figure 4F

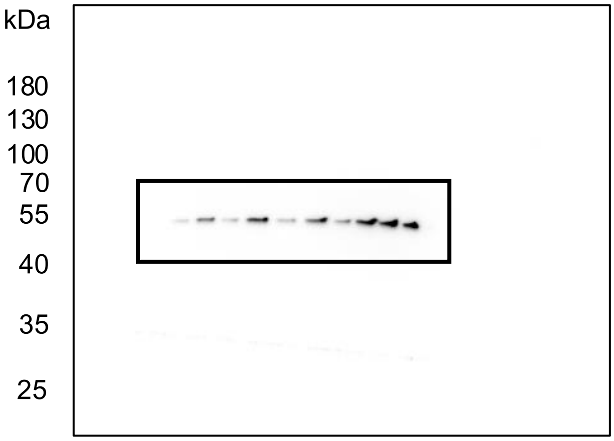

Cyclin A

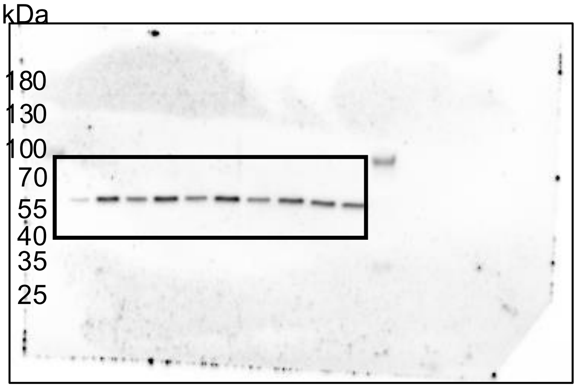

Cyclin E

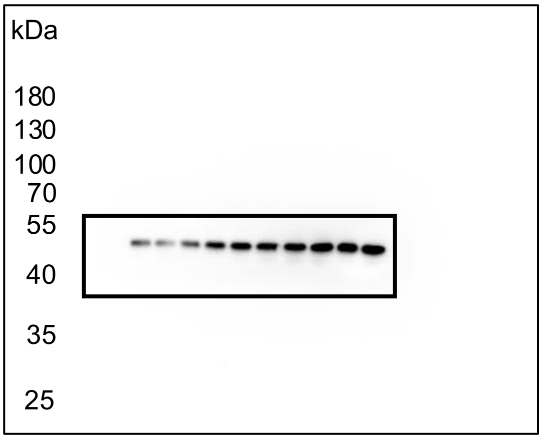

Actin

Figure 4N

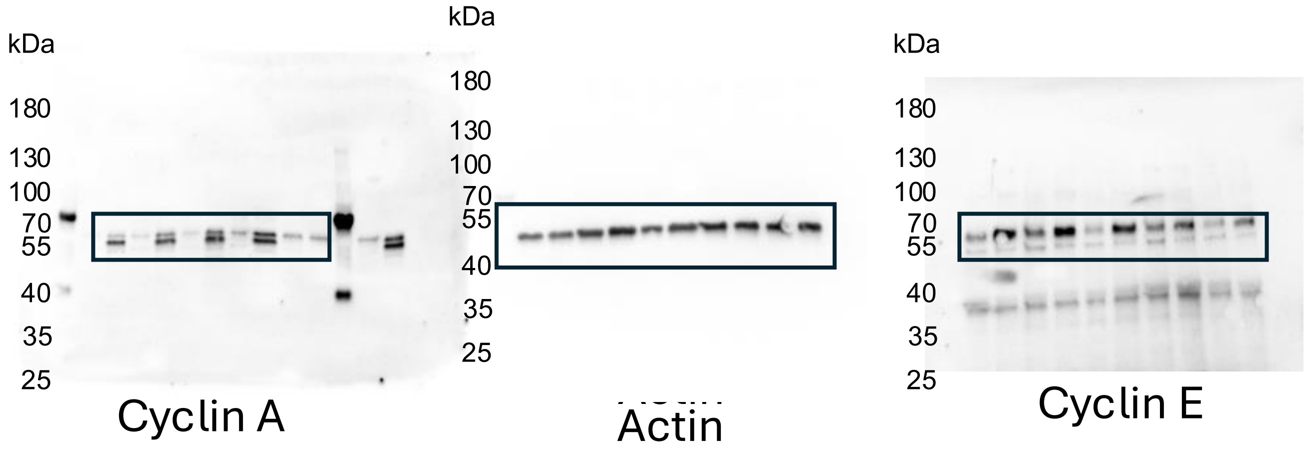

Figure 4O

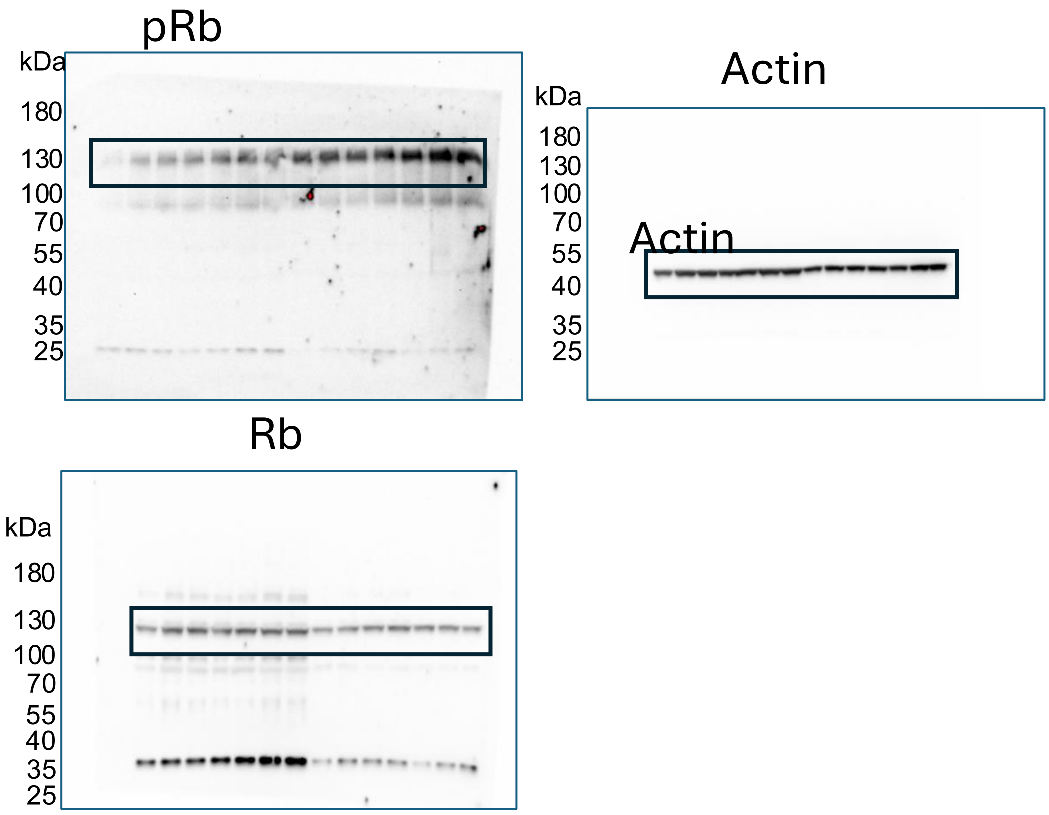

Figure 5E

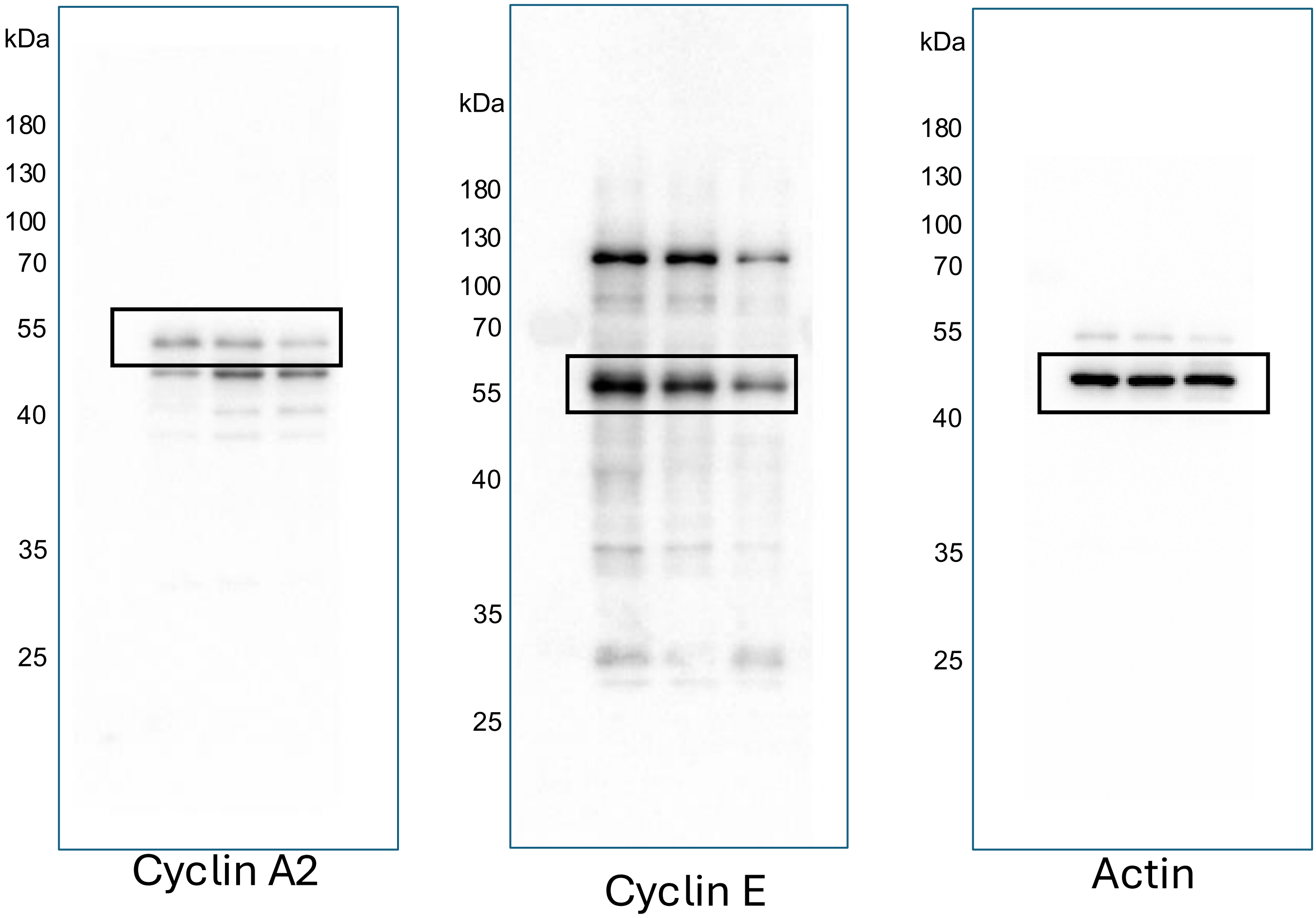
kDa
180
130
100
70
55
40
35
25
Cyclin A2
kDa
180
130
100
70
55
40
35
25
Cyclin E
kDa
180
130
100
70
55
40
35
25
Actin

Figure 5F

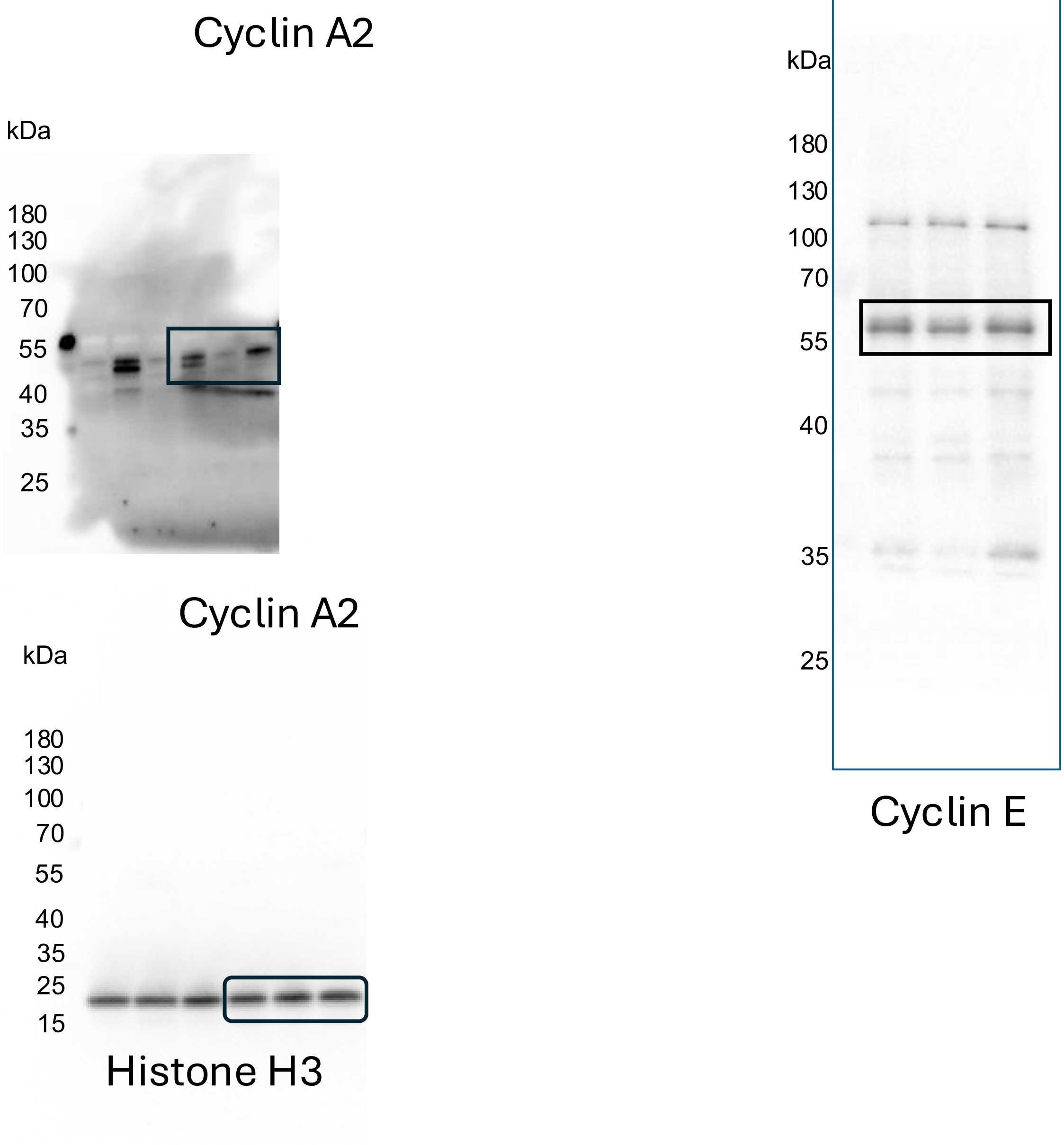
Cyclin A2
kDa
180
130
100
70
55
40
35
25
Cyclin A2
kDa
180
130
100
70
55
40
35
25
15
Histone H3
kDa
180
130
100
70
55
40
35
25
Cyclin E

Figure 6C

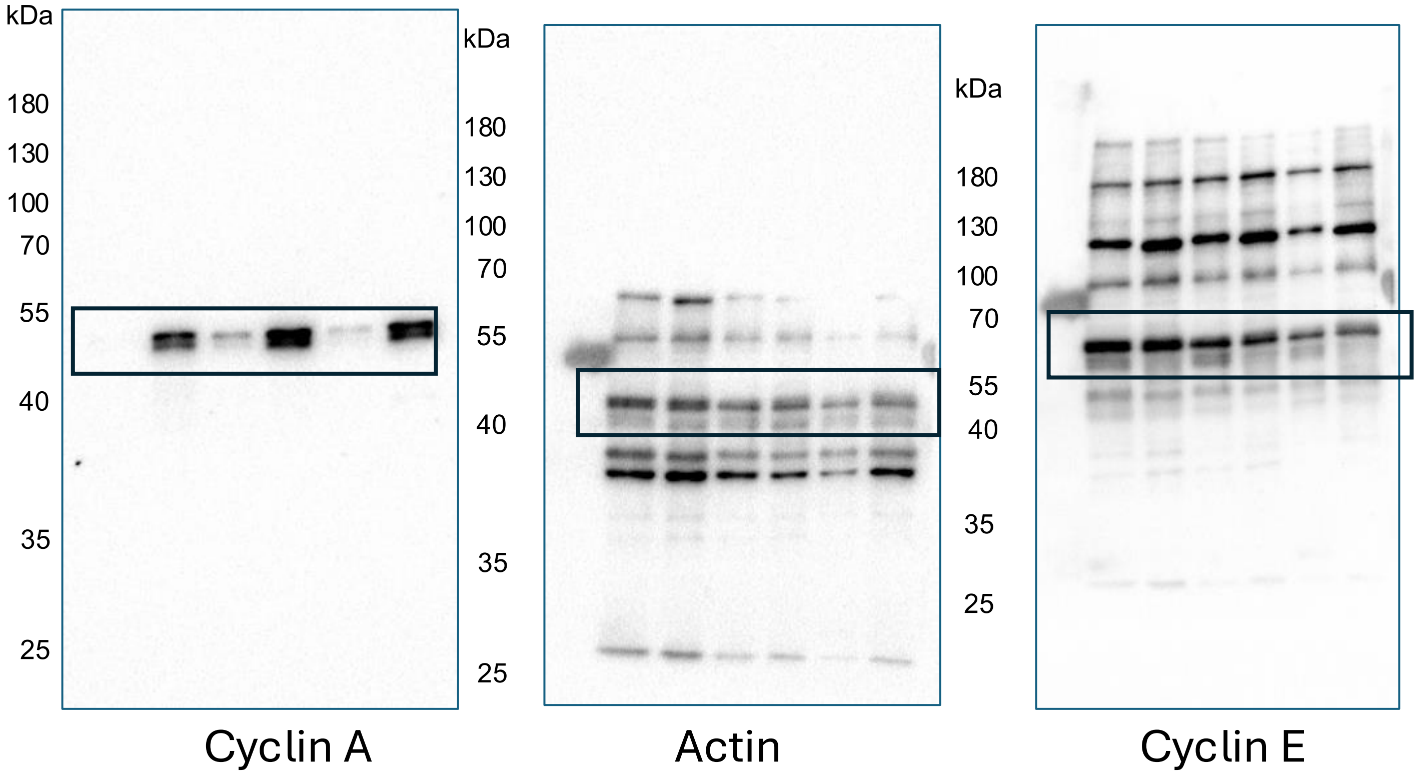

Figure 6D

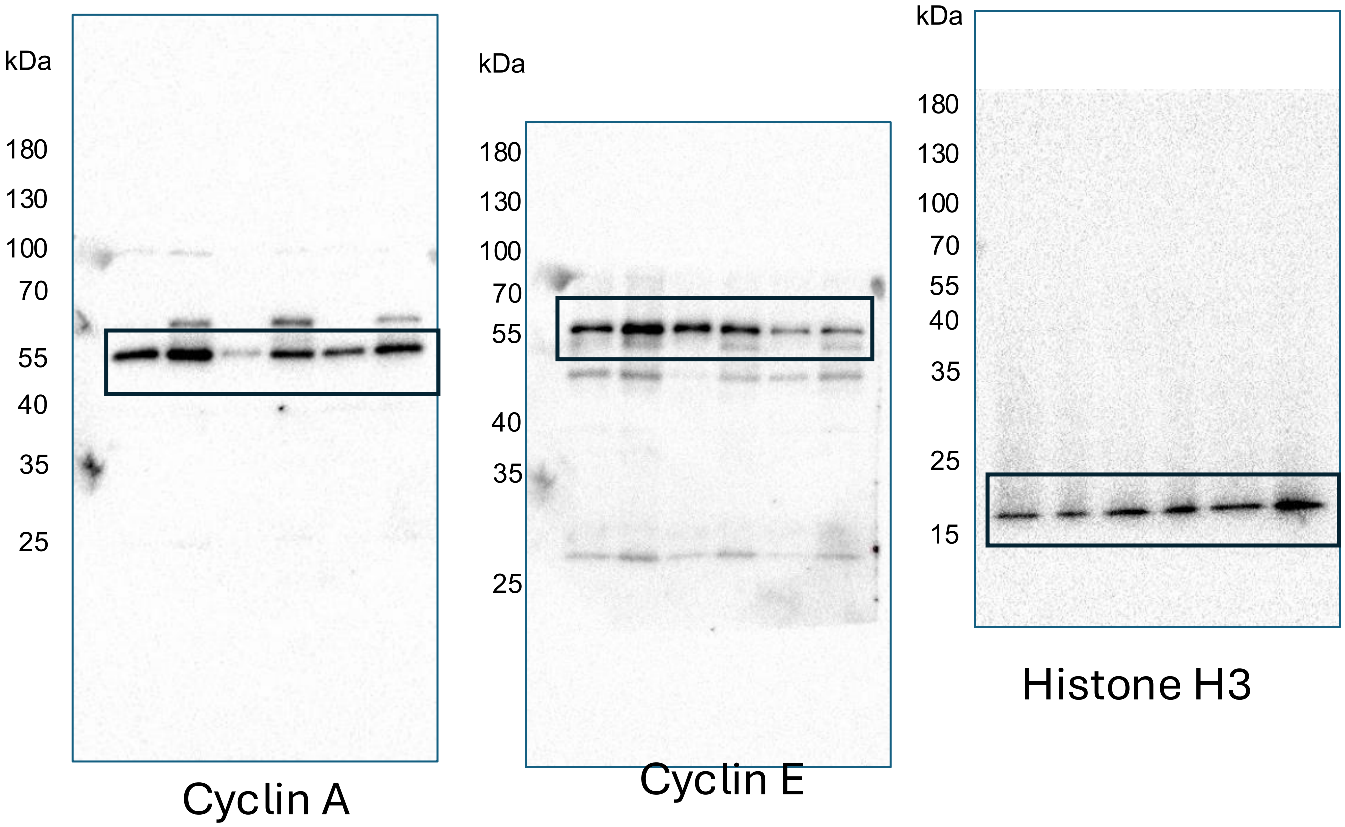

Figure 7e

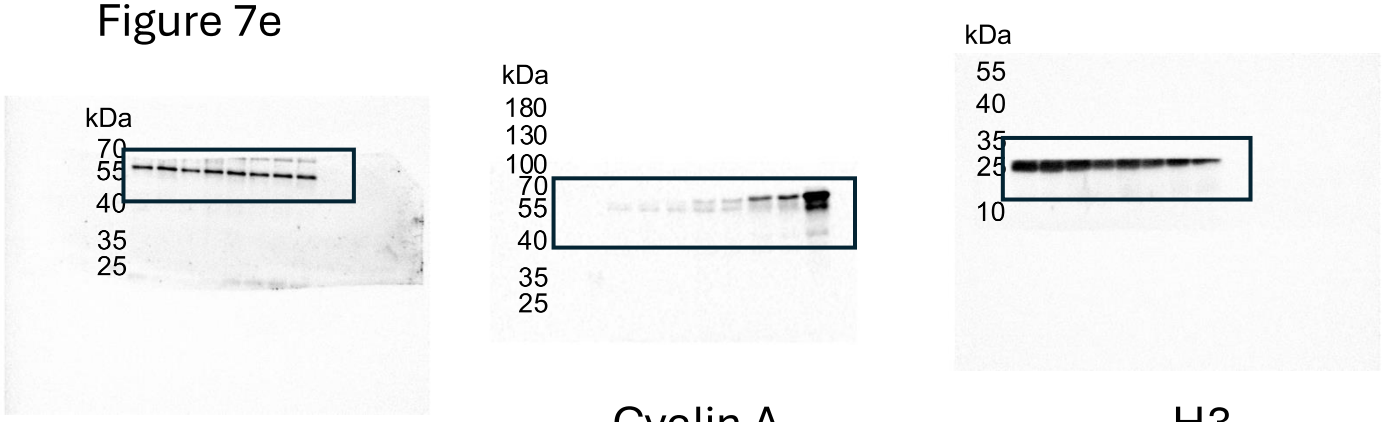

Cyclin A H3

Figure 7f

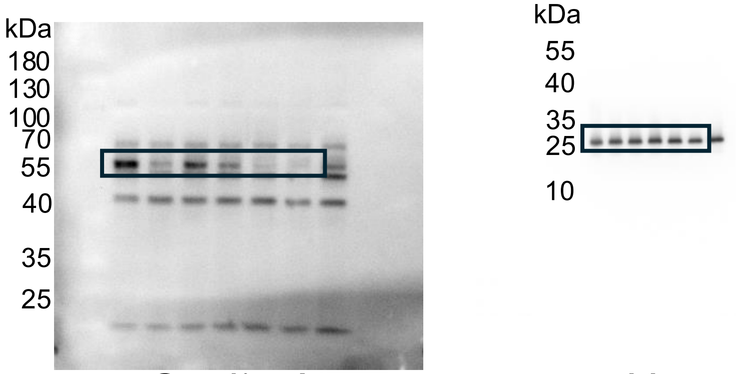

Cyclin A H3

Figure S4J

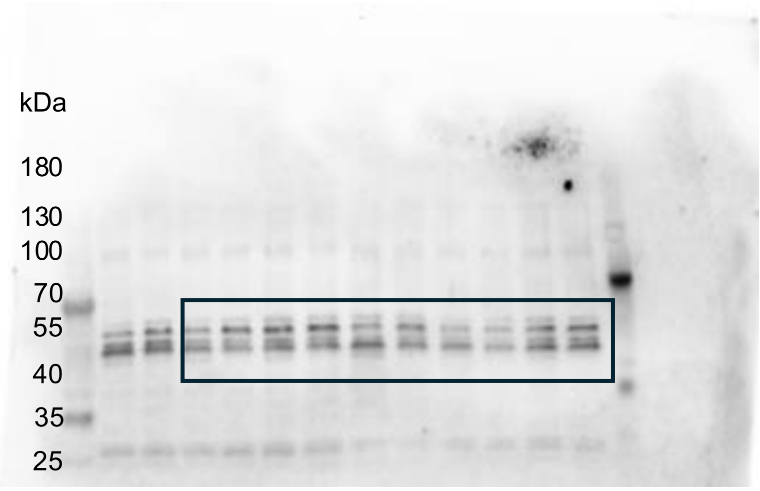

Cyclin E

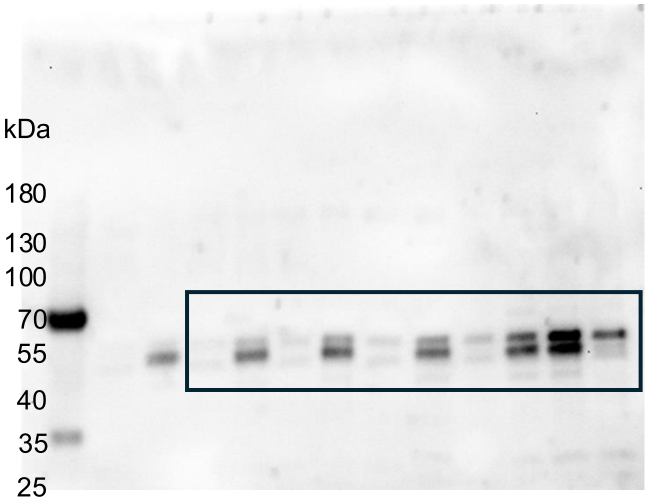

Cyclin A

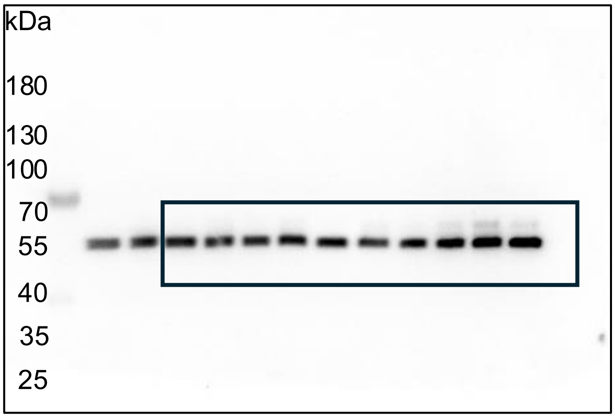

Actin

Figure S4K

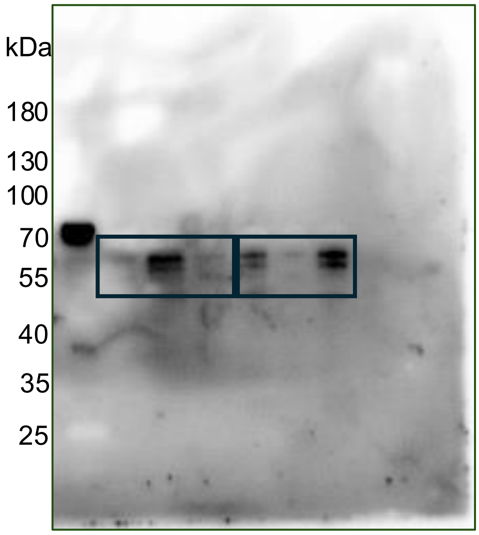

Cyclin A

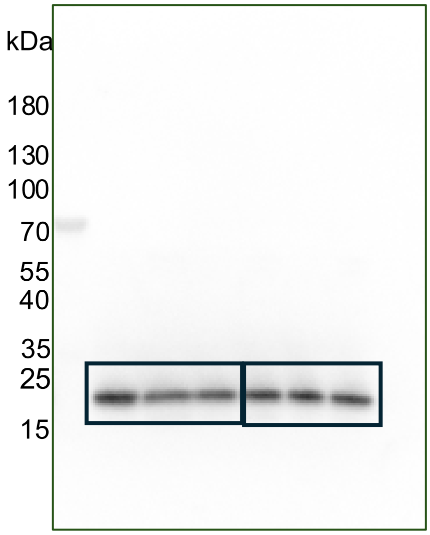

Histone 3

Figure S5 full western blots

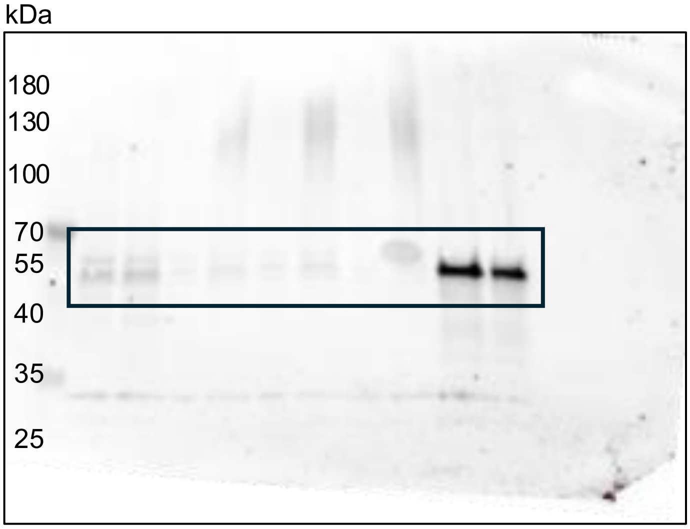

Cyclin A

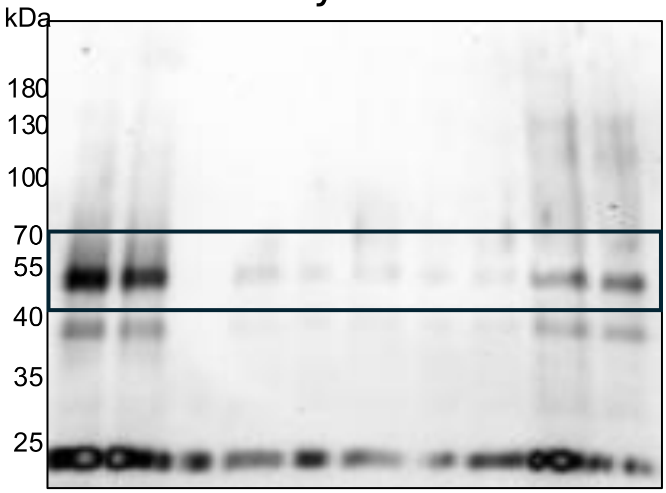

Cyclin E

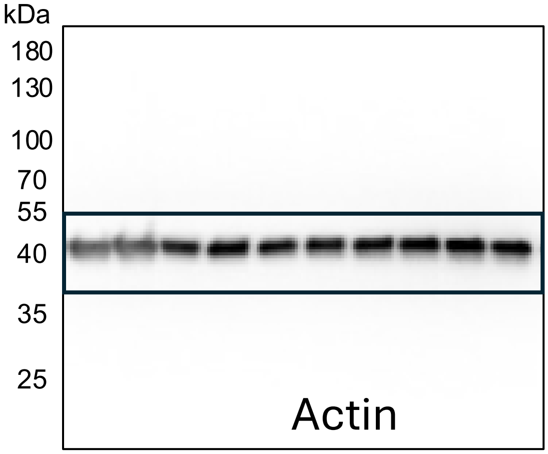

Figure S6 C full western blots

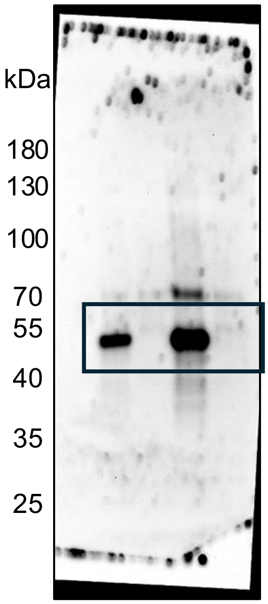

Cyclin A

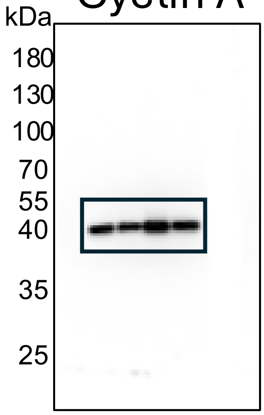

Actin

Figure S6 D full western blots

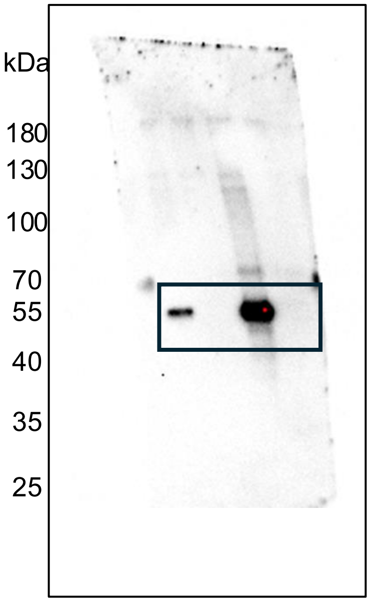

Cyclin E

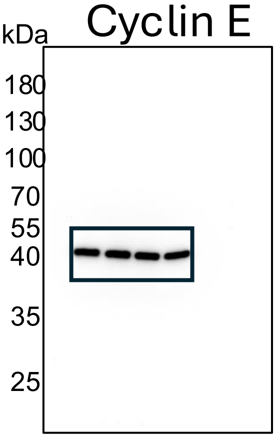

Actin
